# Dopamine-Conjugated Bovine Serum Albumin Nanoparticles Containing pH-Responsive Catechol-V(III) Coordination for In Vitro and In Vivo Drug Delivery

**DOI:** 10.1021/acs.biomac.3c00363

**Published:** 2023-07-14

**Authors:** Eda Argitekin, Esra Ersoz-Gulseven, Gulcin Cakan-Akdogan, Yasar Akdogan

**Affiliations:** † Materials Science and Engineering Department, Izmir Institute of Technology, Izmir 35433, Turkey; ‡ Izmir Biomedicine and Genome Center, Izmir 35340, Turkey; § Department of Medical Biology, Faculty of Medicine, Dokuz Eylul University, Izmir 35340, Turkey

## Abstract

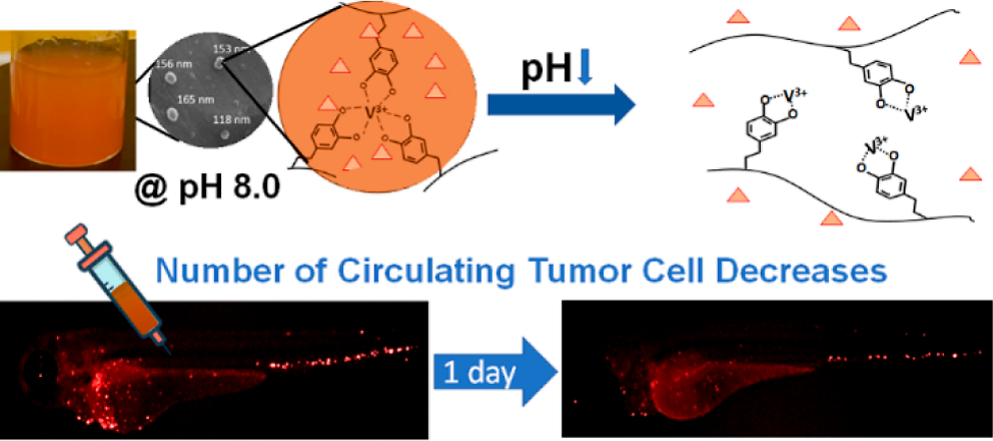

V(III) instead of commonly used Fe(III) provided a rich tris-catechol-metal coordination at pH 7.4, which is important for slow drug release at physiological pH. Bovine serum albumin (BSA) functionalized with catechol-containing dopamine (D) and cross-linked using tris-catechol-V(III) coordination yielded pH-responsive compact D-BSA NPs (253 nm). However, conversion to bis- and/or mono-catechol-V(III) complexes in an acidic medium resulted in degradation of NPs and rapid release of doxorubicin (DOX). It was shown that D-BSA NPs entered cancerous MCF-7 cells (66%) more efficiently than non-cancerous HEK293T (33%) in 3 h. Also, DOX-loaded NPs reduced cell viability of MCF-7 by 75% and induced apoptosis in a majority of cells after 24 h. Biodegradability and lack of hemolytic activity were shown in vitro, whereas a lack of toxicity was shown in histological sections of zebrafish. Furthermore, 30% of circulating tumor cells in vasculature in 24 h were killed by DOX-loaded NPs shown with the zebrafish CTC xenograft model.

## 1 Introduction

Nanoparticle (NP) drug delivery systems not only provide the release of drugs to the targeted areas but also ensure that the drug is used at the required dose and in a sustained release.^1^ Drug release from NPs may depend on the simple diffusion mechanism or it can be activated by a stimulus, e.g., pH variation, temperature, electromagnetic radiation, ultrasound, or electric field.^2−8^ The preparation of pH-sensitive NPs has become a major focus of interest for use in drug release due to the pH difference between the environments of cancer and healthy cells.^9,10^ The pH value of extracellular areas where cancerous tissues are found is in the range of 6.4–6.8, which is acidic compared to that of healthy tissues, pH 7.4.^11−13^ This could be explained by the production of higher amount of lactic acid from cancer tissues.^14,15^ Moreover, the pH values of intracellular endosomes and lysosomes are lower than the pH value of the blood. While the pH value of blood is 7.4, the pH decreases to 6.5 in endosomes and below 5.0 in lysosomes.^16,17^ Therefore, drug-loaded pH-responsive NPs can be rapidly degraded in these organelles, causing rapid drug release.

Serum albumin proteins are widely used to prepare biocompatible drug nanocarriers due to their high capacity of binding for both hydrophilic and hydrophobic drugs.^18−21^ The desolvation method has been widely used in preparation of serum albumin NPs. First, serum albumins are denatured with water-miscible organic solvents, and then cross-linked with glutaraldehyde via the Schiff base bond, which is a pH-sensitive bond.^22−24^ Yang et al. showed that doxorubicin (DOX) release rate from bovine serum albumin (BSA) NPs is faster at pH 5.0 compared to that of at pH 7.4 due to the breaking of the Schiff base bond under acidic conditions.^24^ The cumulative release amounts of DOX for 24 h were found to be 30, 55, and 70% at pH 7.4, 6.5, and 5.0, respectively. Although, glutaraldehyde provides stable NPs with a uniform size distribution, it has toxic potential and ability to interact with drugs in the same way with proteins, which is undesirable.^25,26^ Therefore, as an alternative cross-linking mechanism, Hebel et al. prepared catechol-Fe(III) coordination-mediated human serum albumin (HSA) NPs and hydrogels.^27^ Depending on the catechol/Fe(III) ratio and the pH, HSA NPs between the sizes of 19 to 27 nm were obtained. A well-known catechol-Fe(III) bidentate binding has three pH-dependent stages called as mono-, bis-, and tris-coordinations.^28^ At acidic pH (<5.5), mono-coordination predominates while bis- and tris-coordination are more dominant at above pH 6.0 and 9.0, respectively. Therefore, compact nanostructures could be obtained only at higher pH values with tris-arrangements. In nature, pH-dependent catechol-Fe(III) coordination has been observed in mussel byssal threads.^29^ Kim et al. used recombinant mussel foot proteins (Mfp-1) to form DOX-loaded NPs with the help of Fe(III).^30^ Drug release after 8 h was found to be around 80% in the pH 6.0 medium where mono- and bis-coordination are present. Yet, the cumulative release was found to be 50% at pH 7.4 where only bis-coordination is present.

Reduction of drug release can be achieved at physiological pH, if the tris-catechol-metal cross-linking is maintained at pH 7.4. It has been shown that at pH 7.4 only bis-catechol-Fe(III) coordination was observed but tris-catechol-Fe(III) coordination begins to form above pH 8.5 and predominates at pH 9.0.^28^ However, at the same time, oxidation of catechol to quinone can be observed and causes a non-reversible covalently cross-linked network.^29,31,32^ To achieve tris-catechol-metal complex formation at physiological pH values without catechol oxidation, V(III) ions can be used instead of Fe(III). It has been reported that the tris-catechol-V(III) complex predominates already at pH 8.0.^33^ Therefore, herein V(III) was chosen in order to prepare a nanocarrier with slow drug release under physiological conditions but fast under acidic conditions. First, BSA proteins were functionalized with catechol containing dopamine (D). Desolvated D-BSA proteins were transformed into stable D-BSA NPs in the presence of V(III) as a result of tris-catechol-V(III) complex formation. DOX-loaded D-BSA NPs showed a slow and limited drug release due to the compact structure of D-BSA NPs at pH 7.4. Lowering the pH resulted in degradation of the NPs due to the transition from tris- to bis- and/or mono-coordination, which causes fast and more drug release. The in vitro cellular uptake of the DOX-loaded and unloaded NPs, and their effects on the cell viability were studied using MCF-7 breast cancer cells. In addition, targeting circular cancer cell capacity of NPs and decreasing their numbers by DOX-loaded D-BSA NPs were studied in vivo using zebrafish.

## 2 Experimental Section

### 2.1 Materials

BSA (MW: 66.5 kDa lyophilized powder, >96%), *N*-(3-dimethylaminopropyl)-*N*′-ethylcarbodiimidehydrochloride (EDC), *N*-hydroxysuccinimide (NHS), dopamine hydrochloride, vanadium(III) chloride, phosphate-buffered saline (PBS), albumin-fluorescein isothiocyanate conjugate (FITC), 16-doxyl stearic acid (16-DSA), 4-hydroxy-2,2,6,6-tetramethylpiperidine-1-oxyl (TEMPOL), KOH, acetone, methanol, ethanol, propanol, and neutral-buffered formalin were purchased from Sigma-Aldrich. Acetonitrile, paraformaldehyde, Tween-20, and Triton X-100 were purchased from Merck. Doxorubicin hydrochloride (DOX) was purchased from SelleckChem. All chemicals and solvents were of analytical grade and utilized without any purification procedures. The pH of the solutions was adjusted with hydrochloric acid and sodium hydroxide. Cleaved Caspase-3 (#9664T) and anti-rabbit secondary antibodies (#4412S) were purchased from Cell Signaling Technologies. The normal goat serum was purchased from Diagvonum. Vybrant DiI (V22885) was purchased from Thermo Scientific.

### 2.2 Preparation of Dopamine-Conjugated BSA Protein

**Figure 1:**
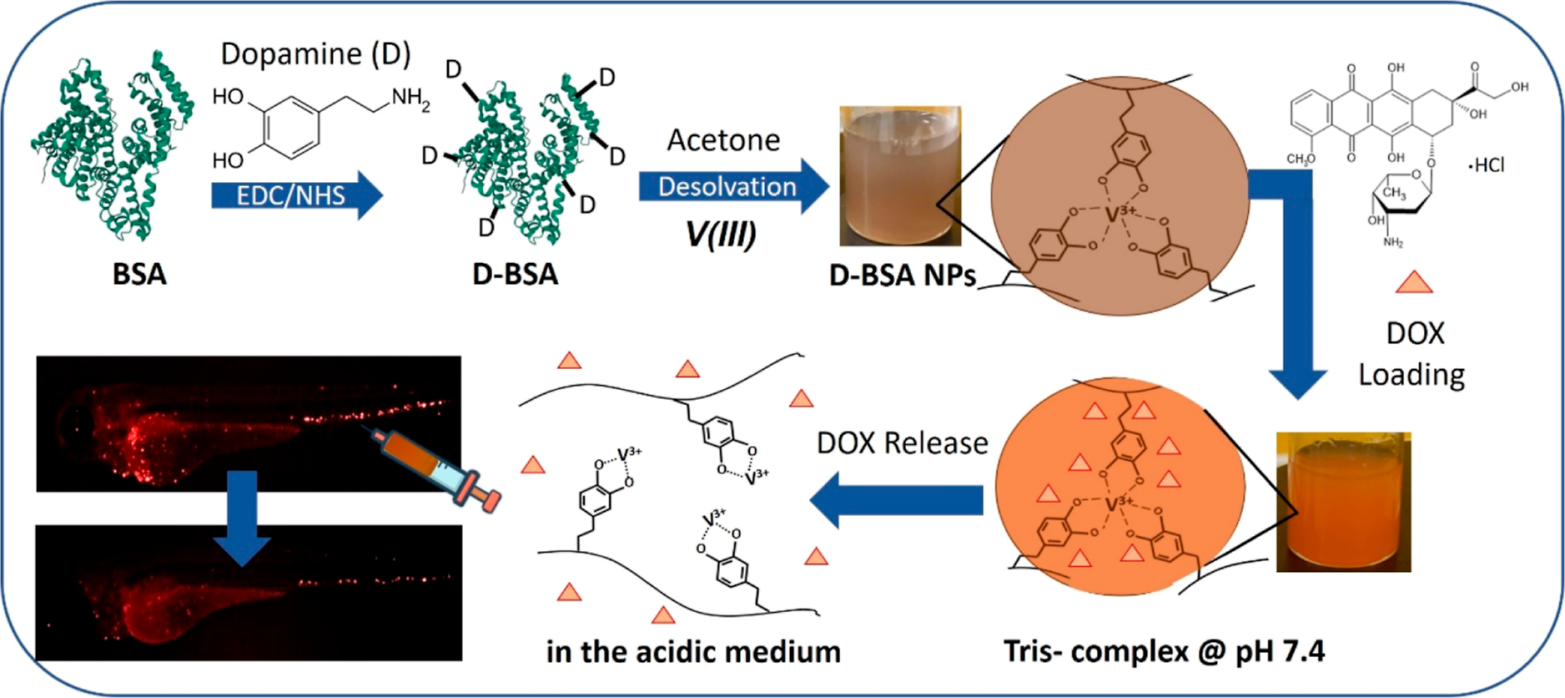
Schematic representation of D-BSA and DOX-loaded D-BSA NPs formations. DOX was loaded on D-BSA NPs containing tris-catechol-V(III) coordination at pH 7.4, and DOX was released in the acidic medium upon degradation of NPs via formation of mono-catechol-V(III) coordination. Injection of DOX-loaded D-BSA NPs to zebrafish larval vasculature killed circulating tumor cells.

72 mg dopamine hydrochloride was dissolved in 3 mL PBS (0.02 M, pH 7.2) and then was added into BSA solution (72 mg in 3 mL deionized water). They were stirred under argon gas for 15 min at 37 °C. The pH of the mixture was adjusted to 6.0 by adding HCl (1 M). Subsequently, 72 mg EDC and 72 mg NHS were added into the mixture and kept at 37 °C for 2 h under argon gas (Figure 1). The reaction was stopped by the addition of 4 M acetate buffer (4 M, pH 6.0). D-BSA was purified by deionized water for the elimination of excess amount of EDC, NHS, and dopamine hydrochloride through 5 cycles of centrifugation using a centrifugal filter (molecular cut off: 50 kDa) (12,000 rpm, 2 min). The final D-BSA was collected from the centrifugal filter, and then it was stored at 4 °C. The yield of the product was 75 ± 8%.

### 2.3 Preparation of D-BSA NPs

9.5 mg of lyophilized D-BSA was dissolved in 980 μL of pure water for 15 min at 750 rpm stirring. Then, pH of the solution was increased to 7.4 by the addition of 20 μL NaOH (0.1 M). Afterward, 5 mL of acetone/water (4:1) (v/v) was added dropwise to aqueous solution of albumin at a rate of 1 mL/min with a syringe pump and 36 μL of VCl_3_ solution (0.081 M) was added to the protein solution at 1200 rpm. After the addition of VCl_3_, pH of the solution was increased to between 7.4 and 8 by the addition of NaOH (0.5 M) in each 4 min and stirred overnight. Next, the obtained D-BSA NPs were transported to Eppendorf tubes in order to be centrifuged. Unbound albumins, excess solvents and VCl_3_ solution were removed by centrifuging the NPs at 12,000 rpm for 15 min. The supernatants were removed from the Eppendorf and the obtained pellets were washed with one time methanol/water (1:1) (v/v) and two times methanol/water (1:3) (v/v) for the purification of the NPs. The yield of the product was 65 ± 5%.

### 2.4 Characterization of D-BSA Protein and D-BSA NPs

Zeta potential of the D-BSA and BSA proteins were analyzed by a Malvern dynamic light scattering (DLS) Nano-ZS instrument (Worcestershire, UK). Molecular masses of D-BSA and BSA were determined by a mass spectrometer Bruker Autoflex-III (smartbeam) MALDI TOF/TOF system. Catechol groups on modified BSA was determined by a LAMBDA 365 UV–vis spectrophotometer (PerkinElmer). Dried protein samples were analyzed by an attenuated total reflectance Fourier transform infrared (ATR-FTIR) spectrometer (Thermo Scientific Nicolet iS50).

Conformational and antioxidant studies of BSA and D-BSA proteins were performed with a CMS 8400 (Adani) benchtop X-band electron paramagnetic resonance (EPR) spectrometer at room temperature. 26 mM 16-doxyl stearic acid (16-DSA) was prepared in 0.1 M KOH and mixed with different concentrations of BSA or D-BSA to get a final 16-DSA concentration of 1.5 mM.

Protein/16-DSA molar ratios were set to 1:2, 1:4, and 1:7. Antioxidant studies were carried out using 1.4 mM TEMPOL prepared in 0.01 M PBS buffer at pH 7.4. TEMPOL solution was mixed with 1.4 mM BSA or D-BSA protein solution prepared in the same PBS solution with a 1:1 (v/v) ratio, and the mixture of TEMPOL and proteins were measured with an EPR spectrometer immediately and after 24 h. Simulations of EPR spectra were done using a Matlab based Easyspin 4.5.5 software package to obtain the rotational correlation time of TEMPOL.^34^

For D-BSA NP characterization, purified NPs were dissolved in distilled water at pH 7.4. Scanning electron microscopy (SEM) was used to examine the size and shape of NPs (SEM, FEI QUANTA 250 FEG). Dissolved NPs were diluted three times with distilled water. 4.5 μL solutions of NPs were dropped onto aluminum foil and dried for 1 day. The dried samples were then coated with gold in a vacuum using an EMITECH K550X for SEM imaging. The accelerating voltages ranged between 5 and 7 kV. In addition, the size of NPs was measured using a Malvern DLS at a wavelength of 632 nm. The scattering angle was set at 173°. Malvern DLS Nano-ZS instrument (Worcestershire, UK) was used to calculate the zeta potentials of NPs. For the size and zeta potential measurements, dissolved NPs were diluted 10 times with distilled water. Vanadium content of D-BSA NPs was determined with an Agilent 7850 inductively coupled plasma mass spectroscopy (ICP-MS). Before the analysis, the sample was digested by using 4% HNO_3_ solution at 180 °C with CEM MARS 6 (microwave accelerated reaction system). The X-ray diffraction (XRD) experiment was carried out with a high-resolution Philips X’PertPro X-ray diffractometer. The fixed divergence slit size is 0.76 mm. The diffraction data were collected with a scanning speed of 0.2°/min in between 2θ = 15 and 70°.

### 2.5 DOX Loading to D-BSA NPs

For DOX loading, the incubation method was used. 3.1 mg of synthesized D-BSA NPs was dissolved in 375 μL PBS (0.005 M, pH 7.4). 15.5 μL of DOX-HCl (43 mM) was added drop-by-drop on the NP suspension and they were stirred overnight, 300 rpm in the dark. The final DOX/D-BSA molar ratio is 15:1. DOX-incubated D-BSA NPs were transported to Eppendorf tubes in order to be centrifuged at 12,000 rpm for 15 min. For the calibration curve for unbound DOX, different amounts of DOX-HCl were added drop-by-drop on PBS (0.005 M, pH 7.4) without D-BSA NPs. Stirring conditions and the centrifuge conditions were the same with incubation. Supernatants were measured by using a LAMBDA 365 UV–vis spectrophotometer (PerkinElmer). The calibration curve was linear and passed through the origin (*R*^2^ = 0.9988, *n* = 5). Entrapment efficiency and drug loading were determined by formulae 1 and 2, respectively.
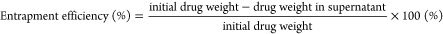
1
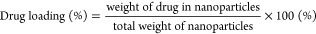
2

### 2.6 In Vitro Release Studies of DOX from D-BSA NPs

In vitro release of DOX from D-BSA NPs prepared with DOX/D-BSA NPs (molar ratios 15:1) was performed in 0.01 M PBS buffer at pH 7.4, 5.5, and 4.2. DOX-loaded D-BSA NPs (1.9 mg) were dispersed in 800 μL of 0.01 M PBS buffer at pH 7.4. Then, the solutions were transferred in 800 μL D-tube dialyzers (Merck, MWCO 3.5 kDa). The dialyzer tube was placed in a beaker containing 32 mL of PBS buffer at pH 7.4 at 37 °C under stirring at 500 rpm. At predetermined time points, 2 mL of sample was collected and measured by a UV–vis spectrophotometer to determine the released amount of DOX from D-BSA NPs. After measurements, the 2 mL of samples were put back in the beakers. The measurements were repeated in PBS media at pH 5.5 and 4.2.

### 2.7 Biodegradation Test

The protocol for biodegradability testing of HSA NPs reported by Langer et al.^35^ was followed for the enzymatic degradation of D-BSA NPs. 1000 μg/mL of D-BSA NPs suspended in 0.005 M PBS buffer at pH 7.4 was mixed with trypsin enzymes. Final concentrations of trypsin were adjusted to 50 μg/mL.^35^ The degradation of NPs upon trypsin addition was determined using turbidity results of the suspension. The suspension was mixed with 500 rpm at 37 °C and the turbidity was obtained photometrically at 565 nm after different time intervals. For the control experiment, the same protocol without trypsin was applied to D-BSA NPs. The remaining NPs during the experiments were determined by the calibration curve obtained from D-BSA NPs at different concentrations: 240, 320, 420, 550, 750, and 1000 μg/mL.

### 2.8 Hemolysis Assay

Red blood cells were isolated from mouse blood to test the hemolytic effect of NPs, according to previously published protocols.^36^ 3 mL mouse blood pool generated from 3 animals was obtained from Izmir Biomedicine and Genome Center (IBG) Vivarium with IBG-Local Ethics Committee approval issued on 18.05.2023. The red blood cells (RBCs) were precipitated by centrifugation at 4000 rpm for 15 min, and then the RBCs were resuspended in PBS. 0.2 mL RBC suspension was mixed with 0.8 mL NP dispersions (in PBS) of different dilutions to obtain final NP concentrations of 0.5, 0.025, and 0.0125 mg/mL. The mixture was incubated in a shaker incubator for 4 h at 37 °C and 30 rpm. Deionized water and PBS were used as positive and negative controls, respectively. The mixture was centrifuged for 10 min at 3000 rpm to precipitate intact RBCs and absorbance of supernatant was read at 540 nm. The percentage of hemolysis was determined by formula 3
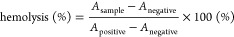
3

### 2.9 Flow Cytometry Assay

1 × 10^6^ cells MCF-7 and HEK293T cells were seeded in a culture plate with Dulbecco’s modified Eagle medium (DMEM) with 10% fetal bovine serum (FBS), 1% penicillin, and 1% streptomycin. FITC-labeled D-BSA NPs and DOX-loaded FITC-labeled D-BSA NPs were suspended in DMEM. The cells were treated with 0.075 mg/mL FITC-labeled D-BSA or DOX-loaded D-BSA NPs for 3 and 24 h. Cells were trypsinized, washed with PBS, and resuspended in PBS for flow cytometry analysis. Untreated and treated cells were scanned with a flow cytometer (BD FACSCanto II), populations of FITC-positive and negative cells were quantified. Experiments were run in duplicate and average values were calculated. Statistical analysis was done with GraphPad Prism 8. Multiple *t*-tests were used for statistical analysis.

### 2.10 Cell Uptake Imaging

NPs in MCF-7 breast cancer cells were imaged with confocal microscopy as described previously.^18^ Cells stained with Dil were seeded at 50,000 cells/well density on 8-well imaging slides. Attached cells were treated with 0.075 mg/mL FITC-labeled D-BSA NPs or 0.075 mg/mL DOX-loaded FITC-labeled D-BSA NPs for 24 h. Excess NPs were removed by washing with 1× PBS-T, and cells were fixed with 4% formaldehyde, and post-stained with DAPI as described previously.^18^ Images were acquired with a Zeiss LSM880 confocal microscope, with 40X/W objective, Z-stacks with 7 μm interval were captured. Background subtraction and maximum Z-projection was applied with ImageJ software.

### 2.11 MTT Assay

MCF-7 or HEK293T cells were seeded 7500 cells/well in 96-well plates and treated with D-BSA NPs (or DOX-loaded D-BSA NPs) at final concentrations of 0.1, 0.05, 0.025, 0.0125, 0.0025, and 0.0005 mg/mL or free DOX at final concentrations of 18, 9, 4.5, 2.25, 0.45, 0.09 μM. After 24 h incubation with the NPs or DOX, the MTT cell viability assay was performed as described previously.^37^ For quantification of formazan, absorbance at 570 nm was measured with a Thermo Fisher Multiskan Go Plate Reader. The decrease in signals was used for calculating cell survival compared to control wells with 100% survival. Each measurement was done in four repetitions, and % viability was calculated.

### 2.12 Cell Death Assay

Cell death induction by apoptosis was tested with immunofluorescence staining of apoptosis marker cleaved-caspase-3 antigen. After 24 h treatment with 0.075 mg/mL NPs, the cells were fixed with 4% formaldehyde for 20 min at RT and washed three times with PBS. Cells were permeabilized with PBS-T (% 0.1 Triton-X100) for 5 min at RT and washed with PBS for 15 min. Cells were blocked with blocking buffer (PBS, 0.1% Tween 20, 5% normal goat serum) for 1 h at RT. Cells were incubated with 1:200 diluted primary antibodies (CST, 9664T) overnight at 4 °C in a humidified chamber. Anti-rabbit Alexa fluor 488-conjugated secondary antibody was used for fluorescence labeling. Cells were imaged at a confocal microscope LSM 880 (Zeiss, Germany). Image processing was performed with ImageJ software.

### 2.13 Zebrafish Experiments

Adult zebrafish were maintained under standard conditions at 28 °C on a 14/10 h light/dark cycle, at the Izmir Biomedicine and Genome Center Zebrafish Facility. Embryos were collected within 1 h of fertilization, incubated in the E3 embryo medium (5 mM NaCl, 0.17 mM KCl, 0.33 mM CaCl_2_, 0.33 mM MgCl_2_, and 1% methylene blue) at 28 °C in a dark incubator. Larvae were incubated at 34 °C after xenograft injection. Experiments were done according to national ethics regulations and in accordance with EU Directive 2010/63/EU, larvae up-to five days post-fertilization were used, which do not require an ethics permit.

The circulating tumor cell (CTC) xenograft model was generated as described previously.^37^ Approximately 100 cells were injected into the duct of Cuvier of the larvae at 2 dpf, and NPs were injected 1 day post-cell injection (dpi). Approximately 1 nL of NP suspension (in water) of FITC-labeled D-BSA NPs or DOX-loaded FITC-labeled D-BSA NPs were injected. For quantitation of cell numbers, larvae were imaged with a fluorescent stereomicroscope 1 h and 1 day after injection. Images of each larva were recorded, and number of circulating cancer cells were counted as described previously was used.^37^ Analysis was performed on 10 larvae per group. Statistical analyses were performed using GraphPad Prism. Two-way analysis of variance (ANOVA) was used to compare multiple groups. Multiple *t*-tests were used for the comparison of two groups.

### 2.14 Histopathology of Zebrafish Xenografts

Zebrafish larvae were fixed with 10% neutral buffered formalin overnight at 4 °C, which is processed for paraffin embedding with a series of alcohol and xylene washing steps.^38^ 1 μm sections were obtained with a microtome. Hematoxylin and eosin (H&E) staining was applied, and samples were imaged with a light microscope.

## 3 Results and Discussion

### 3.1 Synthesis and Characterization of D-BSA Protein

**Figure 2:**
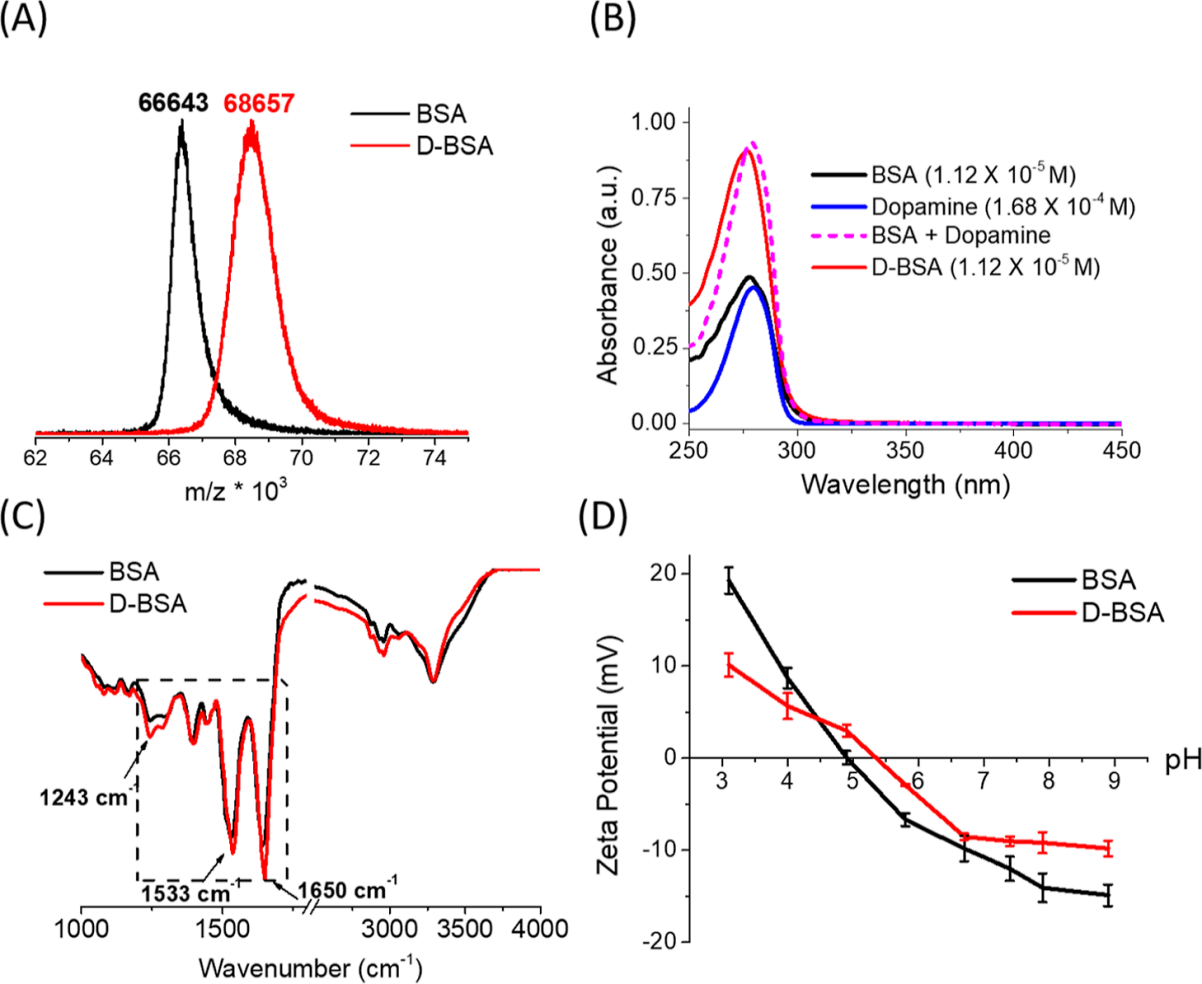
(A) MALDI-TOF mass spectra of BSA (black) and dopamine-conjugated BSA (D-BSA) (red) proteins. (B) UV–vis absorption spectra of 1.12 × 10^–5^ M BSA (black), 1.68 × 10^–4^ M dopamine (blue), sum of BSA and dopamine (×15 mol) spectra (pink), and 1.12 × 10^–5^ M D-BSA (red). Dopamine concentration is 15 times that of BSA. (C) ATR-FTIR spectra of BSA (black) and D-BSA (red). (D) Zeta potentials of BSA (black) and D-BSA (red) aqueous solutions at different pH values from 3.0 to 9.0.

Here, we functionalized BSA proteins with catechol-containing dopamine (D) molecules to obtain dopamine-conjugated BSA (D-BSA) in the presence of coupling agent of EDC. MALDI TOF mass spectrum shows that the molecular weight of BSA increases from 66,643 to 68,657 g/mol upon conjugation of average 15 dopamines to BSA. (Figure 2A). This number of bound dopamines was obtained by reacting the excess dopamine with the available carboxylic acid side chains of 99 amino acids (Asp and Glu) found in BSA. The weight ratio of dopamine in D-BSA was found to be 3.3%. D-BSA proteins with lower dopamine weight ratios were also obtained, but NP formation could not be achieved using them. Binding of dopamines to BSA can also be detected by UV–vis absorption spectroscopy (Figure 2B). BSA proteins have a broad absorption in a range of 260–300 nm with a maximum at 278 nm due to the presence of aromatic amino acids (tryptophan, tyrosine, and phenylalanine). Also, aromatic dopamine has a similar absorption with a maximum at 280 nm. Since MALDI TOF results show that 15 dopamine molecules bind to each BSA protein, total absorption signals of BSA and 15 dopamines must have a similar intensity with that of D-BSA protein. Figure 2B shows that absorption intensities obtained from BSA–dopamine (×15) mixture and D-BSA are very similar within a few nm shifting.

Dopamine conjugation to BSA was also monitored by ATR-FTIR spectroscopy (Figure 2C). Both spectra of BSA and D-BSA have two sharp signals at 1650 and 1533 cm^–1^ and one weak signal at 1243 cm^–1^ due to the protein peptide bonds called as amides I, II, and III, respectively.^39−41^ Upon dopamine conjugation, a detectable change was observed in the region of amide III at 1243 cm^–1^ due to a new amide bond formation. The surface charges of BSA and D-BSA were monitored via zeta potential measurements in water at different pH values (3.0–9.0) (Figure 2D). For BSA, the zeta potential changed from +20 to −15 mV with an isoelectric point (pI) value of 4.9. On the other hand, the zeta potential of D-BSA varied from +10 to −10 mV with a pI value of 5.3. The pKa values of free dopamine determined in the literature are 8.37, 10.25, and 12.49 for catechol first hydroxyl group, amine group, and catechol second hydroxyl group, respectively.^42^ In the neutral water, hydroxyl groups of dopamines could not be deprotonated, therefore, they do not affect the charge of the BSA. Also, the amine side of dopamine is used in conjugation with the carboxyl side chain of amino acids in BSA via amide bond formation, so it does not affect the charge of BSA. Therefore, upon dopamine conjugation, the negative surface charge of the protein shifts from −12 to −9 mV mainly due to the decrease in the number of free carboxyl groups on the surface of BSA. Also, the zeta potential of dopamine in water was found to be around zero (Figure S1). The hydrodynamic sizes of BSA and D-BSA proteins are shown in Figure S2A. Upon dopamine conjugation, the average hydrodynamic size increased from 4.8 ± 0.5 to 8.3 ± 0.7 nm and the size of D-BSA did not change significantly at pH 4.2, 5.5, and 7.4 (Figure S2B).

**Figure 3:**
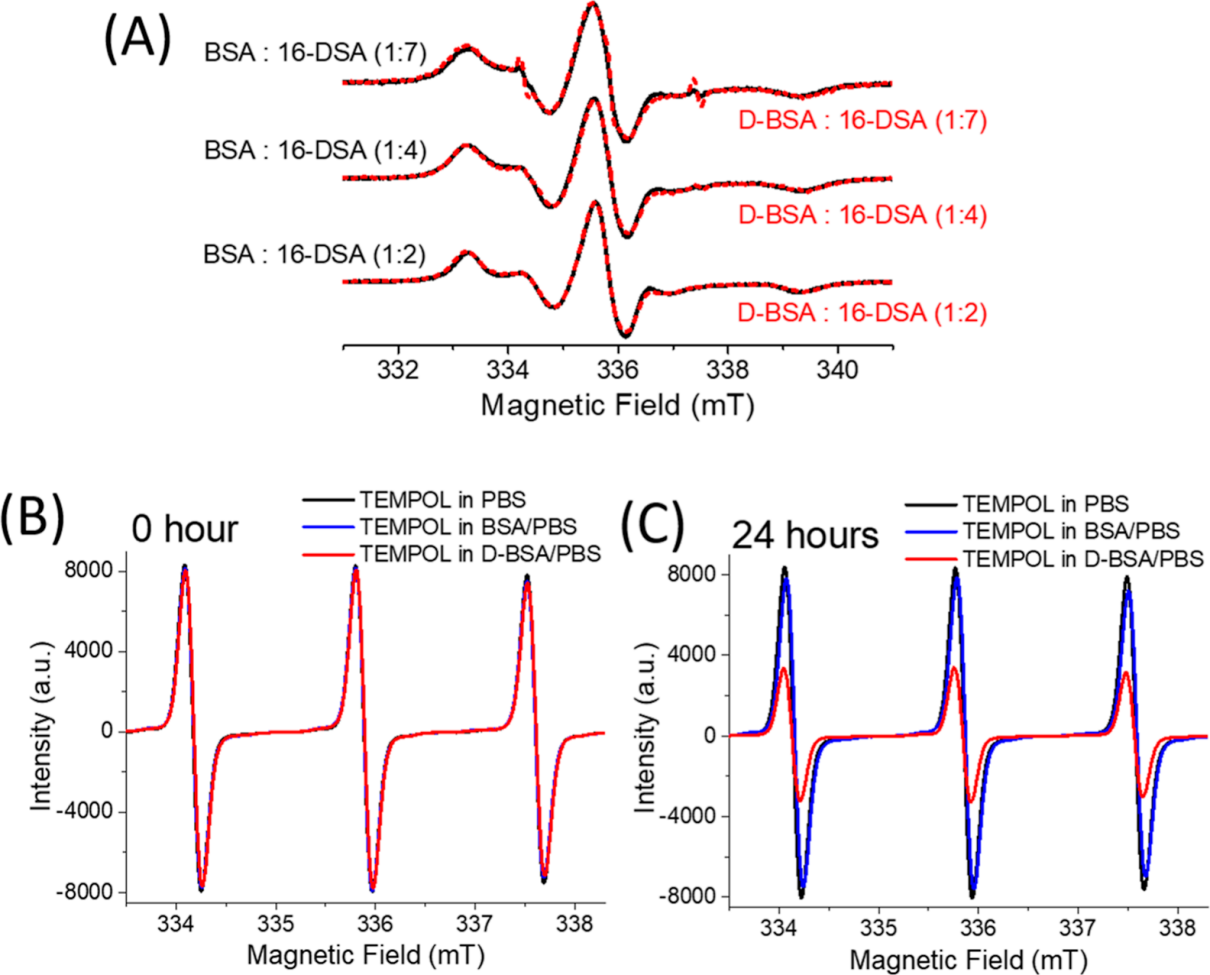
(A) EPR spectra of 16-DSA (1.5 mM) upon binding to BSA (black) and D-BSA (red) at different ratios 1:2, 1:4, and 1:7, BSA (or D-BSA)/16-DSA. (B,C) EPR spectra of TEMPOL (0.7 mM) in PBS (black), BSA/PBS (blue), and D-BSA/PBS (red) just after mixture (B) and after 24 h of mixture (C). BSA (or D-BSA)/TEMPOL ratio is 1:1.

The conformations of BSA and D-BSA can be compared in terms of their fatty acid binding sites. Serum albumin protein has seven fatty acid binding sites and they are often used to study the serum albumin structure by EPR spectroscopy.^43−45^ A conformational change can be recognized by the change in the number of fatty acids bound to serum albumin. Bound and unbound spin-labeled fatty acids can be distinguished by EPR spectroscopy. Bound fatty acids have broad EPR signals due to the restricted rotational motion but unbound fatty acids have sharp EPR signals because of the freely tumbling motion.^43,44^Figure 3A shows that spin-labeled fatty acids, 16-doxyl stearic acid (16-DSA), are bound to binding sites of both BSA and D-BSA, similarly. All broad signals originated from the restricted rotational motion of bound fatty acids were observed up to seven fatty acids per protein. Therefore, it could be concluded that modifying the protein surface with dopamine did not change the fatty acid binding sites of BSA and thus, the conformation of BSA, significantly.

Dopamine have antioxidant features due to its catechol hydroxyl groups.^46−48^ Modification of the BSA surface with dopamine should cause BSA to gain antioxidant properties. EPR spectroscopy can be used to study the antioxidant behavior of BSA surface before and after dopamine conjugation. 4-Hydroxy-2,2,6,6-tetramethylpiperidine-1-oxyl (TEMPOL), which is a nitroxide-based spin radical and can be used to monitor the radical quenching effect of D-BSA. Figure 3B shows that EPR spectra of 0.7 mM TEMPOL in PBS, in BSA/PBS, or in D-BSA/PBS have similar intensities [at BSA (or D-BSA)/TEMPOL ratio is 1:1] just after sample preparation (0 h). However, the EPR signal of TEMPOL decreased by 60% after 24 h in the presence of D-BSA (Figure 3C). On the other hand, addition of BSA did not affect the signal intensity of TEMPOL significantly. These results show that D-BSA has an antioxidant feature, which is not detected for BSA. In addition, the antioxidant property of free dopamine was studied with TEMPOL in PBS buffer. The same amount of dopamine found in D-BSA was mixed with TEMPOL and measured with EPR spectroscopy just after sample preparation (0 h) and after 24 h. The EPR signal of TEMPOL decreased by 43% after 24 h in the presence of free dopamine (data not shown). This indicates that radical quenching properties (antioxidant function) of dopamine is higher when dopamine is conjugated to the BSA protein. Our speculation regarding this phenomenon is that weak interactions between TEMPOL and BSA favor interactions between TEMPOL and dopamines bound to BSA. Figure S3 shows the EPR line shapes of TEMPOL in PBS buffer and in BSA/PBS solutions. The EPR line shape is affected by the rotational dynamics of the radical. Rotational correlation time of a radical increases if the radical is attached to a larger molecule.^49^ Yet, the increase in time depends on the size of the larger molecule and the bond strength between the radical and the larger molecule. Here, the rotational correlation time of TEMPOL, which is affected by the tumbling motion of TEMPOL, increases upon interaction with BSA. A small but detectable increase in rotational correlation time of TEMPOL from 15 ± 6 to 48 ± 10 ps shows the presence of weak interactions between TEMPOL and BSA.

### 3.2 Synthesis and Characterization of D-BSA NPs and DOX Loading

Among metal-catechol cross-linking studies, Fe(III) ion is widely preferred for NPs or hydrogel formation because of its well-known pH-dependent stoichiometry with catechol.^27,28,30^ While bis- and tris-catechol-Fe(III) complexes obtained in the basic medium may lead to the formation of cross-linked nanostructure, the mono-complex obtained in an acidic medium must disassemble the structure. Therefore, initially D-BSA proteins were attempted to be cross-linked with Fe(III) ions to obtain D-BSA NPs. Because tris-catechol-Fe(III) complex formation predominates above pH 9.0, Fe(III) was added to the D-BSA solution at pH 9.0 or at higher pH values.^28^ However, regular NP formation could not be achieved using Fe(III), and this might be explained by the oxidation of catechol side groups to quinone at high pH values, which was detected by the color change of the solution.^29^ Also, addition of Fe(III) ions to the D-BSA protein solution below pH 9.0 did not lead to NP formation. Alternatively, V(III) was used to prepare D-BSA NPs, which can form tris- catechol-metal complexes at relatively lower pH values compared to the pH value for the formation of tris-catechol-Fe(III).^33,50^

D-BSA NPs were prepared by a desolvation method using acetone/water (4:1) mixture followed by the addition of V(III) at pH 7.4–8.0. Addition of a water miscible organic solvent to an albumin aqueous solution called the desolvation method is a thermodynamically driven self-assembly process, which is a common method to prepare albumin NPs.^23,51,52^ Here, a polar aprotic solvent of acetone added to aqueous solution in a dropwise manner under stirring led to turbidity and eventually aggregate formation of D-BSA proteins. The addition of acetone denatures the tertiary structure of albumin proteins, lowering the hydration level of protein as well as the dielectric constant of the solution, resulting in decreased solubility of albumins.^22,53−55^ Increasing the ratio of acetone to water then leads to albumin aggregates, also called coacervate.^56^ However, the resulting particles were not stable enough, so they were converted into stable spherical NPs with a uniform size distribution in the presence of V(III) cross-linker. In order to obtain NPs with a uniform size and shape, several parameters, such as pH, D-BSA and V(III) ion concentrations, acetone/water ratios, stirring speed, and time, were optimized. Other desolvating agents, such as methanol, ethanol, propanol, and acetonitrile were also studied but only the acetone/water (4:1, v/v) mixture caused a high NP formation yield (65 ± 5%).

**Figure 4:**
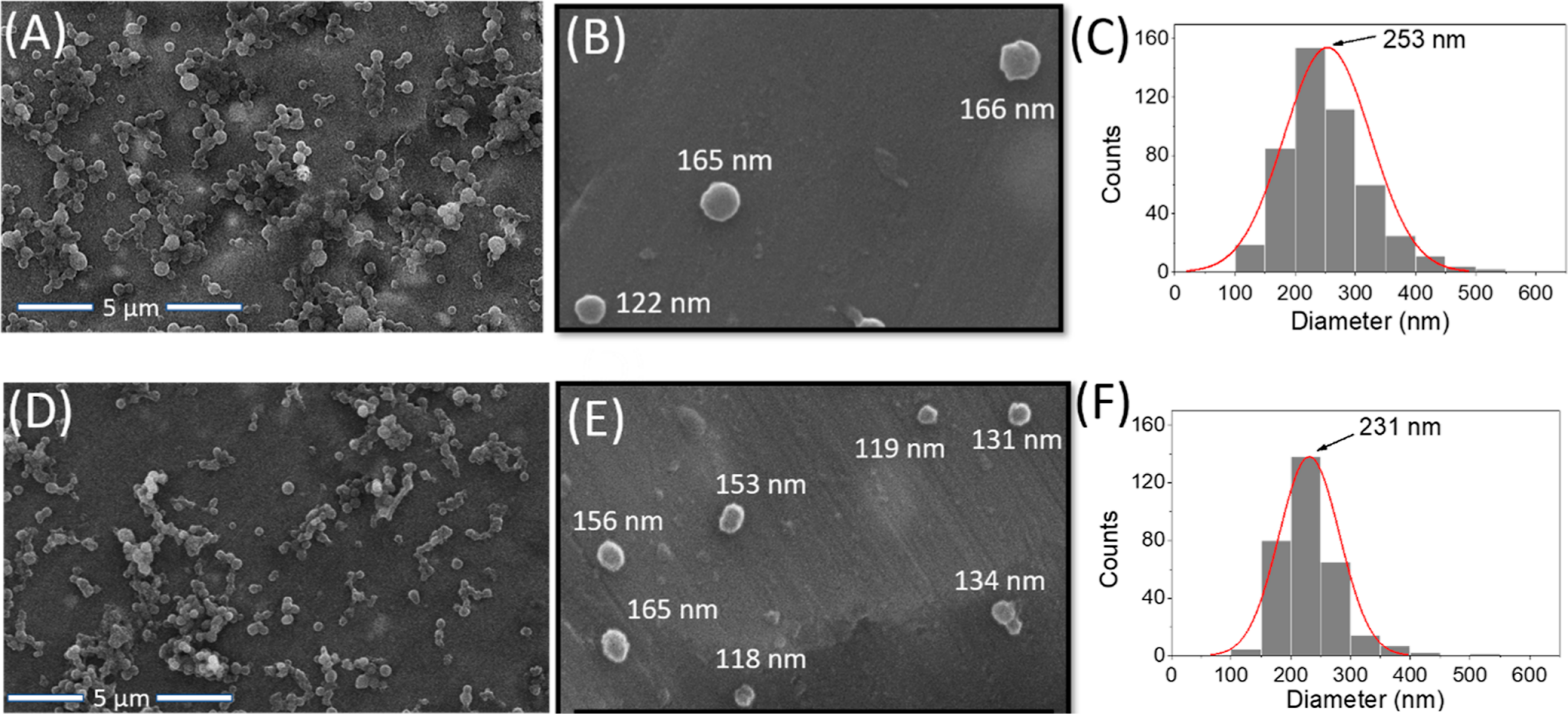
(A,B) SEM images of D-BSA NPs and (C) particle size distribution obtained from the SEM image (A). (D,E) SEM images of DOX-loaded D-BSA NPs and (F) particle size distribution obtained from the SEM image (D).

XRD results of D-BSA NPs cross-linked by catechol-V(III) coordination showed a weak broad signal between 15 and 40° (2θ) which originated from the amorphous structure of NPs (Figure S4).^57,58^ However, aggregates of D-BSA proteins upon acetone addition decomposed in water without V(III)-based cross-linking. The pictures of samples obtained with the cross-linker of V(III) and without V(III) are also shown in Figure S5. Figure 4A,B show the SEM images of spherical D-BSA NPs. Particle size distribution obtained from the SEM image (Figure 4A) shows 253 nm average size with a size distribution between 100 and 400 nm (Figure 4C).

**Figure 5:**
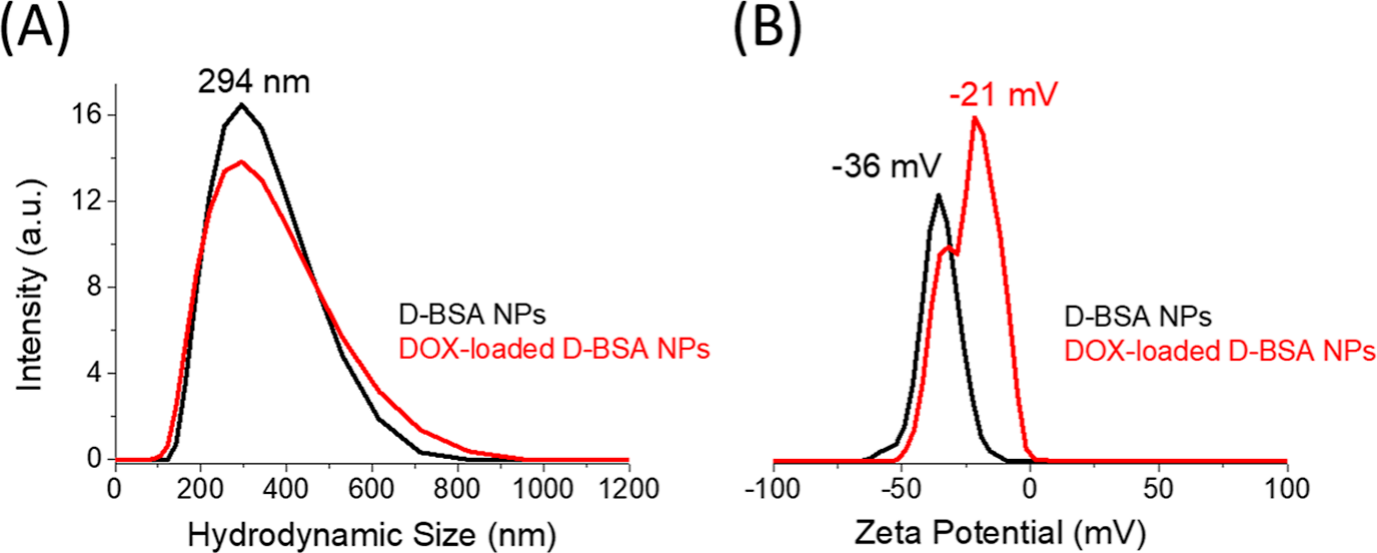
(A) DLS results of hydrodynamic size distributions and (B) zeta potentials of D-BSA NPs (black) and DOX-loaded D-BSA NPs (red) in water. PDI values are 0.15 and 0.16 for D-BSA NPs and DOX-loaded D-BSA NPs, respectively.

In water, hydrodynamic size of D-BSA NPs was obtained with DLS measurements (Figure 5A). The hydrodynamic size distribution of NPs was found to be between 180 and 600 nm with a maximum peak at 294 ± 3 nm. The polydispersity index (PDI) was 0.15 ± 0.01 indicating a narrow size distribution. The reason of higher result obtained from the DLS measurement can be explained by measuring the hydrodynamic size of NPs instead of dried particle size (SEM) and/or swelling of protein nanoparticles with water.^59,60^

**Figure 6:**
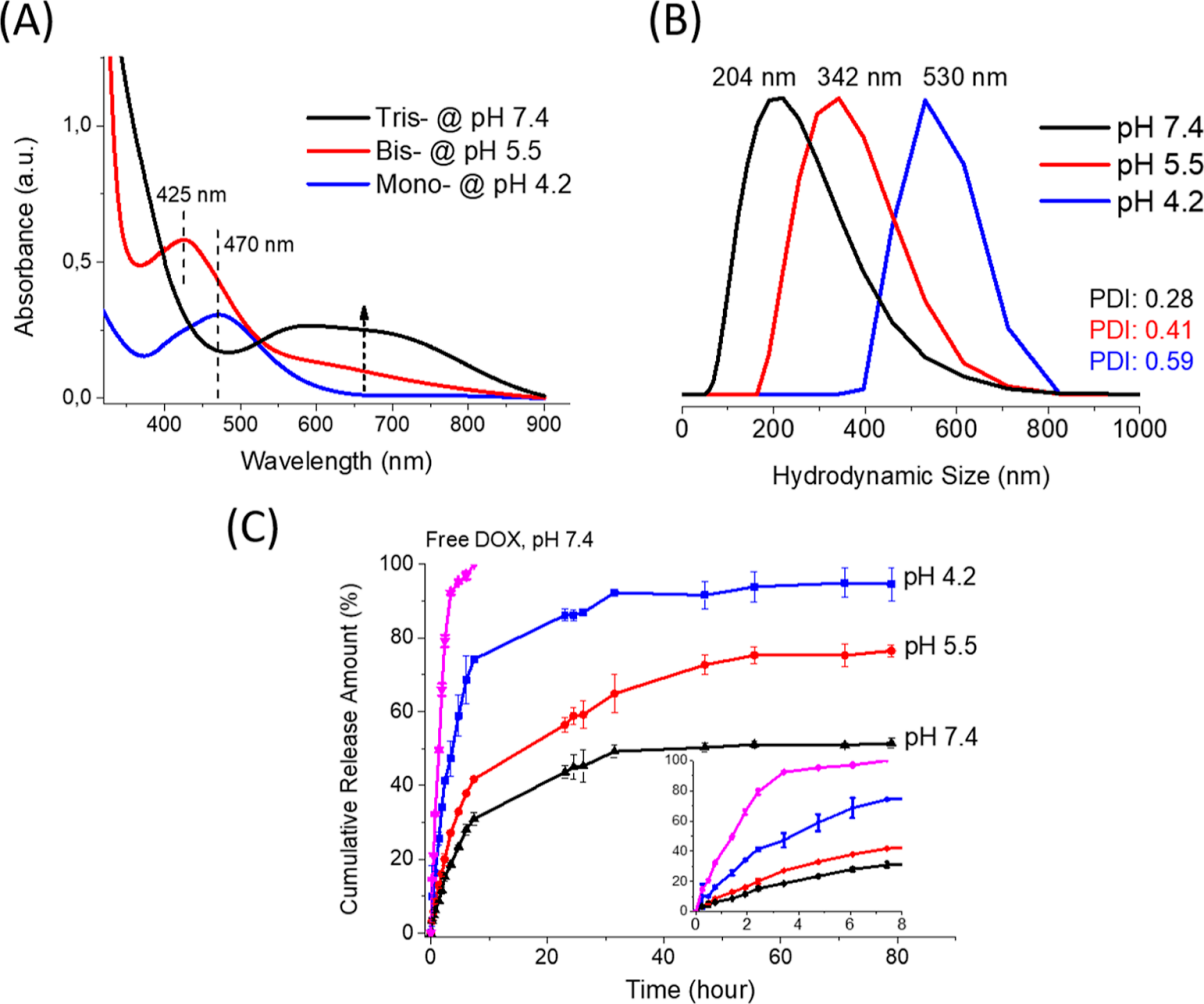
(A) UV–vis absorption spectra of dopamine and V(III) mixture at different pH values: 7.4 (black), 5.5 (red), and 4.2 (blue) under argon gas. (B) DLS results of hydrodynamic size distributions of D-BSA NPs in PBS buffer at different pH values: 7.4 (black), 5.5 (red), and 4.2 (blue). PDI values are 0.28, 0.41, and 0.59 for pH (black), 5.5 (red), and 4.2 (blue), respectively. (C) Cumulative release amount of DOX from D-BSA NPs in 0.01 M PBS buffer at 37 °C at pH 7.4 (black), pH 5.5 (red), and pH 4.2 (blue). As a reference measurement, the release rate of the same amount of free DOX was also performed (pink). The initial 8 h of DOX release amounts from all samples were displayed in the inset of figure (C).

The drug loading capacity of D-BSA NPs was studied with an anticancer drug doxorubicin (DOX). DOX loading can be achieved in two different ways. Drugs can be incorporated into the protein solution during the desolvation process (NP formation) or drugs can be incubated with the obtained NPs in water. Both methods were applied, but it was observed that a high drug concentration ratio, e.g., 15:1 (DOX/D-BSA) prevents the NP formation during the desolvation process. Therefore, first D-BSA NPs were prepared and then water-soluble DOX·HCl was loaded to the D-BSA NPs using the incubation method. For a 15:1 DOX/D-BSA molar ratio, DOX encapsulation efficiency and drug loading capacity of D-BSA NPs were found to be 98 and 10%, respectively. The color of D-BSA NP pellets changed from brown to orange upon DOX loading (Figure 1). SEM images and DLS results show that DOX loading does not cause a significant change in D-BSA NP size (Figures 4D–F and 5A). Because DOX has a hydrophobic aromatic base and polar hydroxyl and amino groups, both hydrophobic and hydrophilic interactions should play a role in its binding to NPs. Upon the high drug loading (10%), the maximum zeta potential of D-BSA NP surface shifted from −36 to −21 mV (Figure 5B), which shows the partial surface coverage with drugs. The high zeta potential of the NP surface reveals that the NP surface has polar properties. Therefore, DOX binding to the surface can be attributed to the polar interactions between DOX (hydroxyl and amine groups) and the surface of NPs. In addition, the interior of the NP, which has a hydrophobic structure, allows the penetration of DOX through hydrophobic interactions between the aromatic base of DOX and proteins. Therefore, drugs loaded inside the NPs showed a sustained slow release at pH 7.4 after a rapid release of surface-bound drugs (Figure 6C).

### 3.3 Dispersion and Stability of NPs in Water

D-BSA proteins were aggregated upon addition of acetone into the protein aqueous solution (desolvation). After that, the aggregates were cross-linked via catechol-metal coordination to obtain stable spherical NPs. The tris-catechol-metal coordination is stable at pH 7.4; therefore, spherical NPs are not decomposed in water and they are dispersed in water easily. The colloidal suspension of D-BSA NPs in water was obtained due to the large negative surface potential of the NPs. The surface charge of D-BSA NPs obtained from zeta potential measurements was found to be between −17 and −50 mV with an average and a maximum peak at −36 mV (Figure 5B). The presence of negatively charged amino acids, e.g., aspartic acids and glutamic acids on the surface of NPs provides a polar anionic surface. Therefore, electrostatic repulsive forces between anionic NPs as well as hydration layers around the polar surface of NPs keep them apart and avoid particle agglomeration. Yet, increasing the concentration of NPs above 0.5 mg/mL causes agglomeration with time and subsequent precipitation. However, they can be resuspended within minutes using vortex mixing.

The structural stabilities of D-BSA-, D-BSA NP-, and DOX-loaded D-BSA NPs in water were studied using DLS and zeta potential measurements. In Figure S6, the hydrodynamic sizes and surface potentials of these materials did not change significantly over 3 weeks, indicating the stability of these materials. Only the mean zeta potential value of DOX-loaded D-BSA NPs changed from −25 mV average to −35 mV over time due to possible drug release from the surface of the NPs.

### 3.4 pH Sensitivity of D-BSA NPs and pH-Induced DOX Release from D-BSA NPs

pH sensitivity of D-BSA NPs originated from the pH-responsive catechol-V(III) coordination. In the literature, mostly Fe(III) ion has been used as a cross-linking agent inspired by nature.^28,30,61^ Cohesive and adhesive interactions found in the threads and plaques of mussels depend on the interaction between catechol containing DOPA amino acid and Fe(III) ions supplied from seawater.^29,62^ However, tris-catechol-Fe(III) coordination predominates at above pH 9.0, which may lead to catechol oxidation to quinone.^28,29^ Also, in an alkaline environment quinone polymerization yielded hydrogels through irreversible covalent bonding.^63^ To obtain pH-responsive regular spherical NPs, we used V(III) ions because its tris-catechol complex formation predominates at pH 8.0 before the catechol oxidation. Sever and Wilker showed the conversion of mono-catechol-V(III) complex to bis- and eventually tris-catechol-V(III) complexes by the addition of NaOH using UV–vis absorption spectroscopy.^50^ Additions of 2 equiv NaOH per V(III) ions (the corresponding pH value is about 4.5) yielded mono complexes (425 and 467 nm), 4 equiv NaOH (the corresponding pH value is about 7.0) yielded bis complexes (402 and 635 nm), and 5 equiv NaOH (the corresponding pH value is about 10.0) yielded tris complexes (361, 600, and 650 nm) under an argon gas. However, Holten-Andersen et al. showed that a higher rate of tris-catechol-V(III) coordination was obtained compared to bis- and mono-types at pH 8.0 using Raman spectroscopy.^33^ Therefore, we prepared D-BSA NPs at pH between 7.4 and 8.0 to avoid catechol oxidation, which can be observed in a basic medium, and to ensure tris-catechol-metal coordination.

UV–vis absorption signals can be used to show the formation of tris-, bis-, and mono-complexes at different pH values. However, the continuous absorption signal of BSA aggregates formed in the acetone–water mixture prevented detection of mono-, bis-, and tris-catechol-V(III) coordination signals. Therefore, we repeated the same procedure for NP formation with dopamine molecules and V(III) ions. Dopamine/V(III) were mixed at a 3:1 molar ratio at pH 4.2, 5.5, and 7.4 under argon gas and measured by UV–vis absorption spectroscopy (Figure 6A). The main absorption signal at 470 nm was assigned to the mono-catechol-V(III) complex at pH 4.2. Increasing the pH values, this signal shifts to lower wavelengths of 425 nm and ca. 350 nm at pH 5.5 and 7.4, respectively. Moreover, increasing the pH values to 5.5 and 7.4 yielded new broad signals between 550 and 850 nm, which originated from bis- and tris-coordination.

In order to show the pH effect on the structure of D-BSA NPs, they were dissolved in PBS buffer (0.01 M) at different pH values: 4.2, 5.5, and 7.4. NPs were suspended in the buffered solution overnight and measured by DLS. Decreasing the pH value caused larger structure formation due to the breaking of cross-linking in NPs (Figures 1 and 6B). A pH-sensitive metal coordination bond between catechol and V(III) was converted from tris-coordination to bis- and/or mono-coordination; therefore, the compact NP turned into a more open structure. It was found that the maximum intensity at the particle size distribution was shifted from 204 ± 4 to 342 ± 12 and 530 ± 22 nm in PBS buffer at pH 7.4, 5.5, and 4.2, respectively. Also, lowering the pH of the PBS solution led to a less uniform size distribution; this was noticed by an increase in PDI values from 0.28 ± 0.03 to 0.41 ± 0.05 and 0.59 ± 0.05 at pH 7.4, 5.5 and 4.2, respectively. The zeta potentials of D-BSA NPs were found to be similar at pH 7.4 and 5.5, but the absolute value of zeta potential decreased from −28 ± 2 to −20 ± 2 mV at pH 4.2 due to greater protonation in a more acidic solution.

DLS results of the hydrodynamic size distributions of D-BSA NPs differed when distilled water or 0.01 M PBS buffer were used as the dispersion medium (Figures 5A and 6B). The maximum intensity at the particle size distribution was shifted from 204 ± 4 nm (in PBS) to 294 ± 3 nm (in water). Also, the particle size distribution obtained from the SEM image showed a maximum at 253 nm between DLS results of NPs obtained in water and PBS. Our speculation regarding the dispersion medium effect on the size of NPs is that charges on the surface of NPs are screened in the buffer medium but not in water, and this might change the size of protein-based soft NPs. The high surface charge of D-BSA NPs of −36 mV revealed that anionic groups are concentrated on the surface. The electrostatic repulsion between these anionic groups on the surface may cause swelling of NPs in water. On the other hand, the salt ions in the PBS buffer solution screen out the repulsive interactions between the anionic sidechains of amino acids. Therefore, the surface of NPs might become a more collapsed conformation and give a compact structure, just as with polyelectrolyte polymers.^64^ The effect of dispersion medium on DLS results was also studied with an alternative BSA NP prepared using glutaraldehyde as cross-linker after desolvation with ethanol. The DLS result of BSA NPs measured in water was found to be larger (221 nm) than that in PBS (133 nm), a similar trend was observed for the D-BSA NPs (Figure S7).

The pH sensitivity of D-BSA NPs was also investigated by the release dynamics of DOX from the NPs at different pH values (Figure 6C). Mono- and bis-catechol-V(III) complexes predominate at acidic pH values, so the drug release rate as well as the cumulative amount of released drugs are expected to be higher than that observed at physiological pH, which averages 7.4. In the first 8 h of drug release study, the cumulative DOX releases were found to be 31, 42, and 75%, at pH values 7.4, 5.5, and 4.2, respectively. Also, as a reference measurement, free DOX diffusion measurement at pH 7.4 was performed and 100% of DOX release was observed after 8 h. The limited DOX release (31%) observed at pH 7.4 from D-BSA NPs in the first 8 h is also lower than the DOX release from the recombinant Mfp-1 NPs prepared by DOPA-Fe(III) coordination, which was found to be 50% in the first 8 h at pH 7.4.^30^ This might be explained by the presence of only bis-catechol-Fe(III) coordination at pH 7.4 in Mfp-1 NPs, but D-BSA NPs have tris-catechol-V(III) coordination at pH 7.4.

At the end of 80 h, total DOX releases reached to 51, 76, and 95% at pH values 7.4, 5.5, and 4.2, respectively. The significant increase in drug release (up to 95%) can be explained by the formation of mono-catechol-V(III) complexes at pH 4.2. The difference in the total amounts of releases (51 and 76%, after 80 h) at pH 7.4 and 5.5 can be explained by the conversion of tris complexes to bis complexes at pH 5.5. Therefore, DOX release from D-BSA NPs is limited due to the formation of tris-complex structure under physiological conditions, but rapid release can occur by lowering the pH.

### 3.5 Biodegradability and Hemolytic Effect of D-BSA NPs

**Figure 7:**
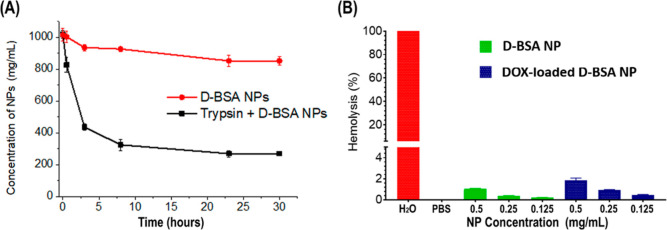
(A) Plot shows the concentration of the D-BSA NPs (mg/mL) over 30 h, in PBS (red line) or in trypsin (50 μg/mL) containing PBS (black). (B) Hemolysis percentage of RBCs quantified with absorbance at 540 nm is plotted after treatment with positive control, H_2_O (100% hemolysis) (red), negative control, PBS (0% hemolysis), 0.5, 0.25, and 0.125 mg/mL D-BSA NPs (green) and DOX-loaded D-BSA NPs (blue).

The biodegradable property of D-BSA NPs was studied with a digestive enzyme of trypsin, which is active under physiological conditions.^35,65^ The suspension of albumin NPs causes turbidity due to their cross-linked structure, which can be detected with UV–vis absorption spectroscopy at 565 or 630 nm.^35,66^Figure S8A shows the calibration curve that was used to calculate the NP concentration from absorption measured at 565 nm. Figure 7A shows that the concentration of D-BSA NPs in the PBS solution decreased over time in the presence of trypsin. After 3 hours, 57% of NPs (1000 μg/mL) were degraded upon interaction with trypsin (50 μg/mL) but only 6% of NPs were degraded in the absence of trypsin at 37 °C (Figure 7A). After 30 h, the degradation reached 73 and 15% in the presence and absence of trypsin, respectively (Figure 7A). This shows that the protein digestive enzyme of trypsin can degrade D-BSA NPs at physiological pH with time.

To test the biocompatibility further, a hemolytic test was performed using murine RBCs. When RBCs were suspended in PBS and incubated with D-BSA NPs at a concentration of 0.5 mg/mL for 4 h under shaking conditions, 1% hemolysis was observed, when normalized to hemolysis caused by distilled H_2_O. When lower concentrations of 0.25 and 0.125 mg/mL were used, hemolysis was close to none. The use of DOX-loaded D-BSA NPs induced slightly higher hemolysis with 0.5 mg/mL causing less than 2% hemolysis (Figure 7B). Figure S8B shows the pictures of the obtained supernatants of positive and negative controls as well as all concentrations tested.

### 3.6 Cellular Uptake of D-BSA NPs

**Figure 8:**
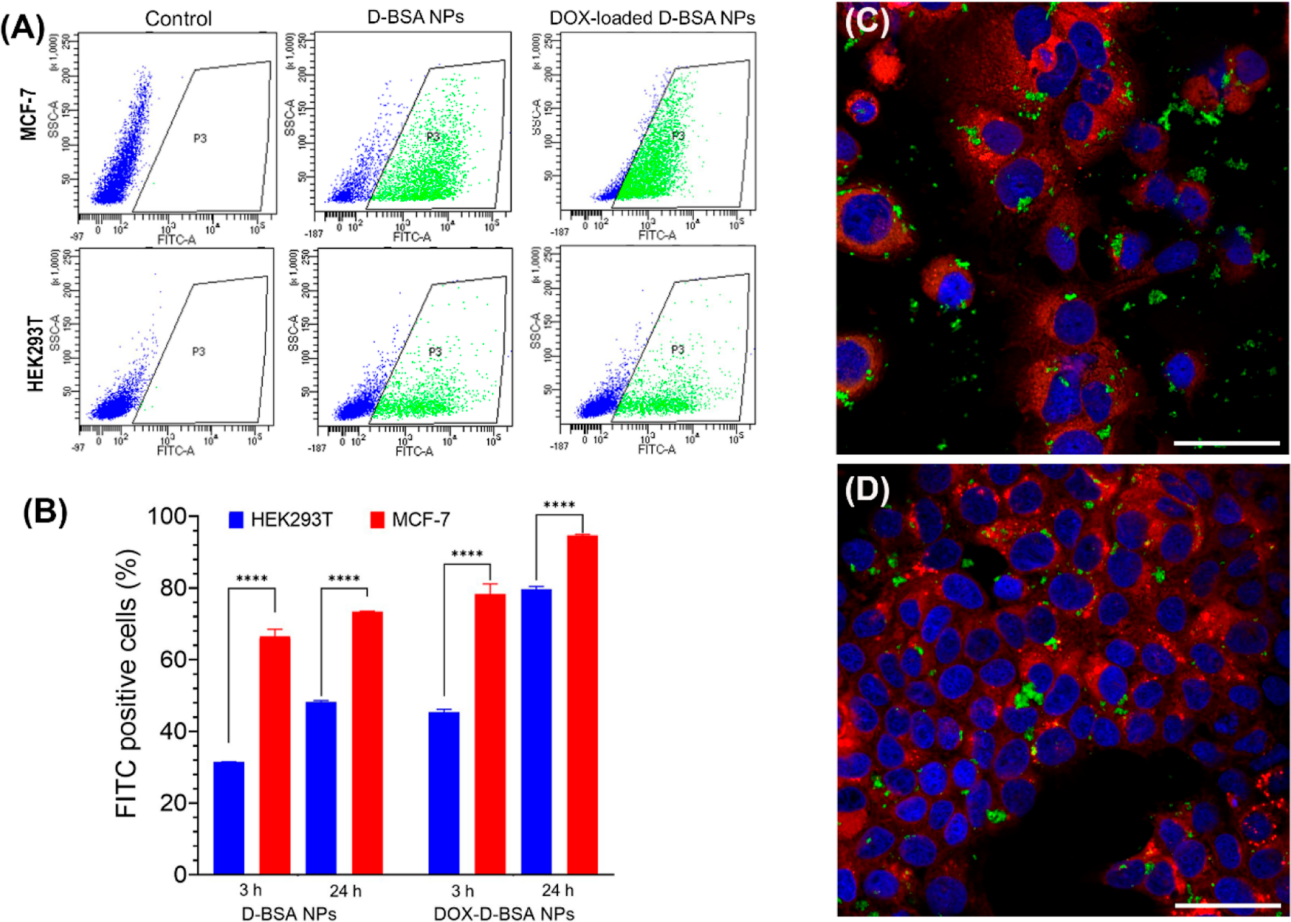
(A) Flow cytometry analysis of cellular uptake assay of FITC-labeled unloaded and loaded NPs. Both MCF-7 and HEK293T cells were treated with NPs for 3 h. Flow cytometry plots show FITC positive cells (green, region P3) and FITC negative cells (blue). (B) Ratio of cells that internalized FITC-labeled NPs were quantified from flow cytometry analysis of 100,000 events and average of two independent cell sets were plotted. NPs were internalized by MCF-7 cells more efficiently compared to HEK293T cells (****p < 0.0001). (C,D) Confocal microscopy images (40XW objective) of MCF-7 cells incubated for 24 h with 0.075 mg/mL of (C) FITC-labeled D-BSA NPs (green) or (D) DOX-loaded FITC-labeled D-BSA NPs (green). Cell membranes were stained with Dil (red) live cell dye and nuclei were stained with DAPI (blue). Scale bar = 50 μm.

Cellular uptake dynamics was studied with the breast cancer cell line, MCF-7, and human embryonic kidney cell line, HEK293T. To this end, fluorescein isothiocyanate (FITC)-labeled BSA proteins were used. FITC-labeled BSA proteins were conjugated with dopamines and then mixed with D-BSA with a 1:4 ratio before the NP preparation. 0.075 mg/mL of DOX-loaded or unloaded FITC-labeled D-BSA NPs were incubated with MCF-7 and HEK293T cells for 3 and 24 h (Figures 8A,B and S9). Flow cytometry analysis showed that when NPs were incubated with MCF-7 cells for 3 h, 66% of cells already have FITC-labeled D-BSA NPs inside, which increased to 73% after 24 h incubation. Interestingly, the uptake of NPs into HEK293T cells was significantly lower with 33 and 48% of cells containing NPs after 3 and 24 h incubations, respectively. Cellular uptakes of DOX-loaded NPs were detected to be slightly higher than the unloaded NPs in all groups; however, the difference between MCF-7 and HEK293T uptakes was still significant (Figures 8A,B, and S9). The uptake of NPs into cancer cells was visualized in MCF-7 cells, after 24 h incubation with 0.075 mg/mL of DOX-loaded or unloaded FITC-labeled D-BSA NPs. It was observed that the NPs were internalized by MCF-7 cells (Figure 8C,D). The mechanism of internalization was not investigated here; however, endocytic or gp60 receptor-mediated uptake of serum albumin NPs has been documented in the literature. Endocytic uptake of negatively charged serum albumin NPs has been shown via both caveolae- or clathrin-mediated endocytosis pathways using inhibitors of filipin and chlorpromazine, respectively.^67,68^ A more preferential cellular uptake of HSA NPs was observed via the caveolae-mediated endocytosis mechanism. Endocytoses of HSA NPs decreased to 44 and 56% when caveolae-mediated and clathrin-mediated endocytoses were blocked, respectively.^67^ Also, glycoprotein 60, gp60, (or albondin), one of the membrane-associated albumin-binding proteins (receptors) and albumin binding proteins known as secreted protein acidic and rich cysteine (SPARC) are involved in the endocytosis of albumin NPs.^69,70^

### 3.7 Effects of D-BSA NPs on Cell Viability In Vitro

**Figure 9:**
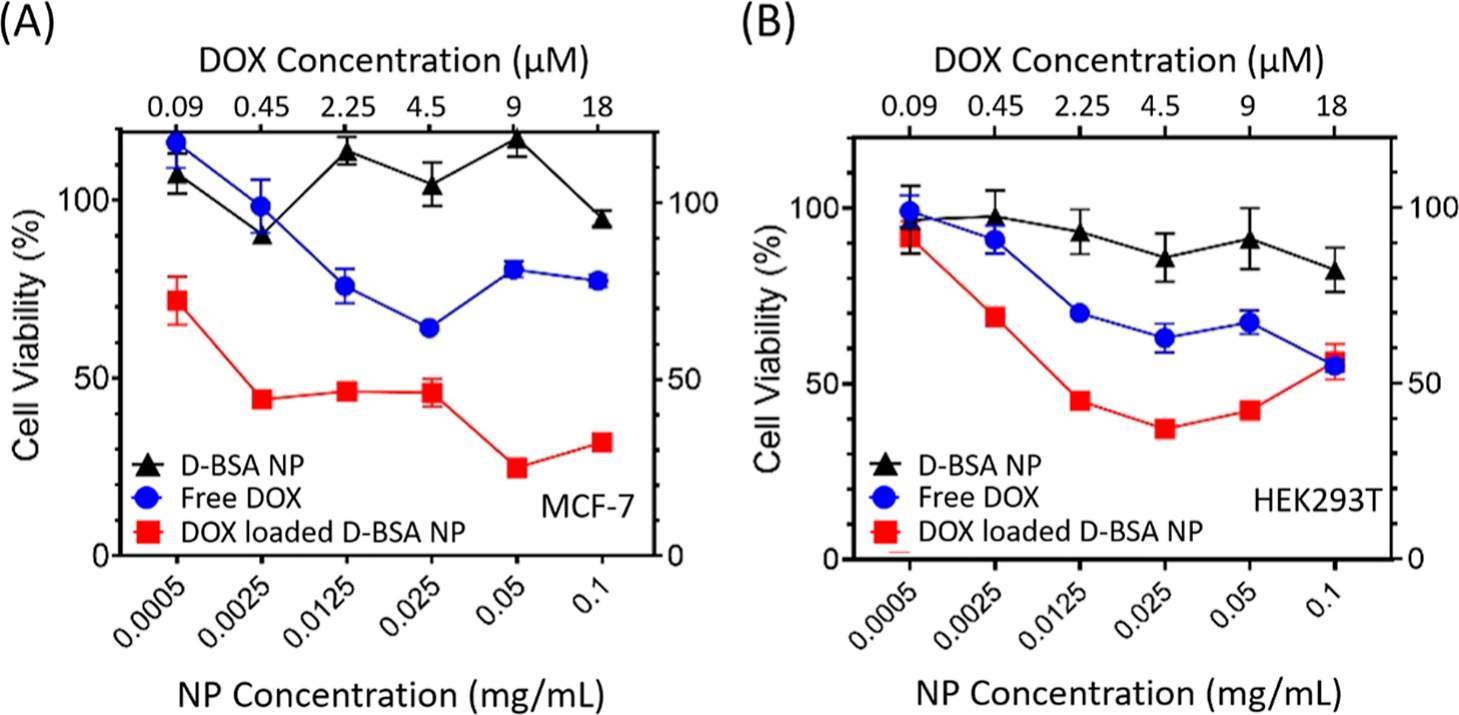
(A) MCF-7 and (B) HEK293T cells were exposed to different dilutions of unloaded (black) and DOX-loaded NPs (red) between 0.0005 and 0.1 mg/mL, or corresponding concentrations of free DOX (blue) for 24 h. Cell viability was displayed as the percentage with respect to untreated control cells.

The cytotoxicity of the prepared D-BSA NPs was tested in both MCF-7 and HEK293T cells with the MTT test after 24 h incubation with NPs. Figure 9 shows that increasing D-BSA concentrations up to 0.1 mg/mL does not affect the viability of MCF-7 or HEK293T cells significantly (black line). The half-maximal inhibitory concentration (IC_50_) value of V(III) ions has been reported to be 70 μM in the literature.^71^ The weight ratio of V(III) in D-BSA NPs was found to be 0.003% by the use of ICP-MS. This shows that only 0.8% of conjugated dopamine is cross-linked with V(III) ions to form tris-catechol-V(III) coordination in D-BSA NPs. As a result, the concentration of V(III) ions used in the formation of tris-catechol-V(III) coordination in 0.1 mg/mL D-BSA NPs was found to be 0.06 μM, which is much lower than the IC_50_ value of V(III) (70 μM).^71^ Therefore, V(III) ions used in the preparation of BSA NPs are not expected to induce any toxicity to cells.

Next, cytotoxicity of DOX-loaded NPs and free DOX was compared in MCF-7 and HEK293T cells. DOX exerts cytotoxicity via the intercalation of DNA, poisoning of topoisomerase-II enzyme, and formation of free radicals that damage DNA, cellular membranes, and proteins.^72^ Both cell lines were sensitive to DOX. 64% of MCF-7 cells and 62% of HEK293T cells were viable after being exposed to 4.5 μM (Figure 9A,B). However, treatment with DOX-loaded D-BSA NPs decreased the cell viability more efficiently at varying concentrations. For example, after treatment with 0.025 mg/mL of DOX-loaded D-BSA NPs (carrying 4.5 μM of DOX), 46 and 37% of MCF-7 and HEK293T cells were viable, respectively. Upon an addition of 0.05 mg/mL of DOX-loaded D-BSA NPs (carrying 9 μM of DOX), the cell viability of MCF-7 decreased to 25%. Above this concentration, cell viability did not change significantly. To sum up, the killing effect of DOX on cells was significantly increased when DOX was given with D-BSA NPs, whereas unloaded D-BSA NPs had no toxicity.

**Figure 10:**
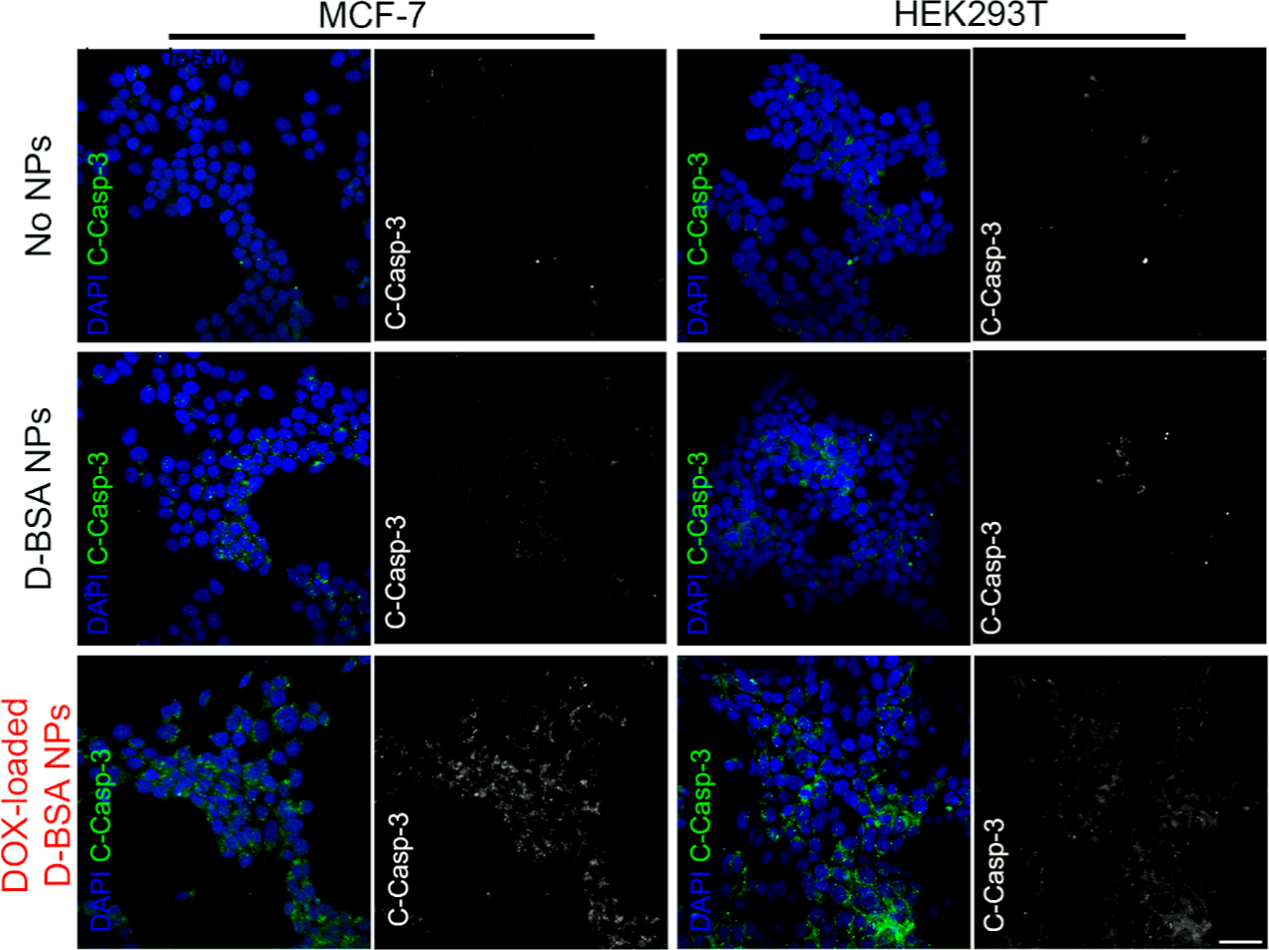
Immunofluorescence staining of C-Casp-3 in MCF-7 (left) and HEK293T (right) cells treated with 0.05 mg/mL D-BSA- and DOX-loaded D-BSA NPs. Untreated cells (top row) were used as the control. Overlay images on the left show a nucleus in blue (stained with DAPI) and C-Casp-3 in green. The C-Casp-3 signal is shown in the grayscale next to the overlay image for each condition. Scale bar: 50 μm.

Cell death induced by D-BSA NPs and DOX-loaded D-BSA NPs was further investigated by immunofluorescence staining of apoptotic marker-cleaved caspase 3 (C-Casp-3) proteins in both cell types. Untreated control cells and cells treated with 0.05 mg/mL NPs for 24 h were fixed and stained with C-Casp-3 antibodies and DAPI to visualize the nuclei of cells. Only few of the untreated and D-BSA NP-treated cells were positive for C-Casp-3, whereas almost all of the cells treated with DOX-loaded D-BSA NPs were positive for C-Casp-3, indicating a strong induction of apoptosis (Figure 10).

### 3.8 CTC Targeting by D-BSA NPs in Zebrafish

**Figure 11:**
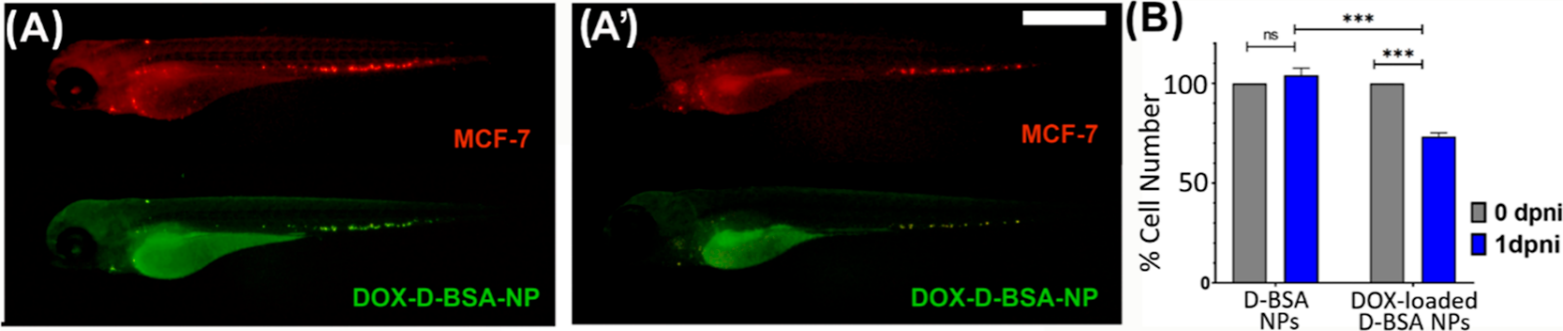
Dil-labeled MCF-7 cells (red) and FITC-labeled D-BSA NPs (green) injected zebrafish larvae. Images were taken at the injection day (0 dpni) (A) and 1 day post-injection (1 dpni) (A′). (B) CTCs at the day of D-BSA NPs and DOX-loaded D-BSA NP injections (0 dpni) and 1 day post-NP injections (1 dpni) were counted in 5 individual larvae and average cell numbers are normalized with their 0 dpni values. Upon 1 day treatment with DOX-loaded D-BSA NPs, number of CTCs decreased by 30% with *p* ≤ 0.001 significance (***), whereas the CTC numbers of D-BSA NP-treated larvae did not change significantly *p* > 0.05). Scale = 500 μm.

A specific albumin receptor called 60 kDa glycoprotein and SPARCs, which are overexpressed by tumor cells, facilitate the uptake of albumin NPs.^73,74^ In addition, albumin NPs are more preferred by tumor cells due to their increased energy and amino acid demands.^75−77^ NPs can also passively accumulate more in the tumor tissues compared to healthy tissues, which is called the enhanced permeability retention effect.^78,79^ Furthermore, NPs can be used to target the CTC, which are more difficult to detect and low in number.^37,80^ CTCs can start distant metastasis and cause a relapse; therefore, it is important to target and eliminate them.^81^ Zebrafish between 2 and 5 days post-fertilization (dpf) provides several advantages, such as transparency, fast organ development, and easy micromanipulation, for detecting and counting CTC in the live organism during the treatment. In a previous study, in vivo CTC targeting capacity of BSA NPs prepared with glutaraldehyde was demonstrated in a zebrafish model.^37^ Here, the cancer targeting efficiency of FITC-labeled D-BSA NPs was tested with the same zebrafish larval CTC xenograft model.^37^ MCF-7 cells were injected into the circulation of 2 dpf zebrafish embryos to generate the xenografts. The next day, D-BSA NPs or DOX-loaded D-BSA NPs were injected, and the xenografted embryos were monitored for 24 h. Ten individual larvae were imaged with a fluorescence stereomicroscope shortly after NP injection and 1 day post-NP injection (1 dpni) (Figure 11A,A′). Cells were outlined and counted using ImageJ cell counter plug-in and average cell numbers for each group was plotted after normalizing them with their 0 dpni values (Figure 11B). While D-BSA NPs did not cause the death of MCF-7 cells in zebrafish, the DOX-loaded D-BSA NPs caused a significant 30% decrease in the MCF-7 cell number within the first 24 h of injection (Figure 11B).

**Figure 12:**
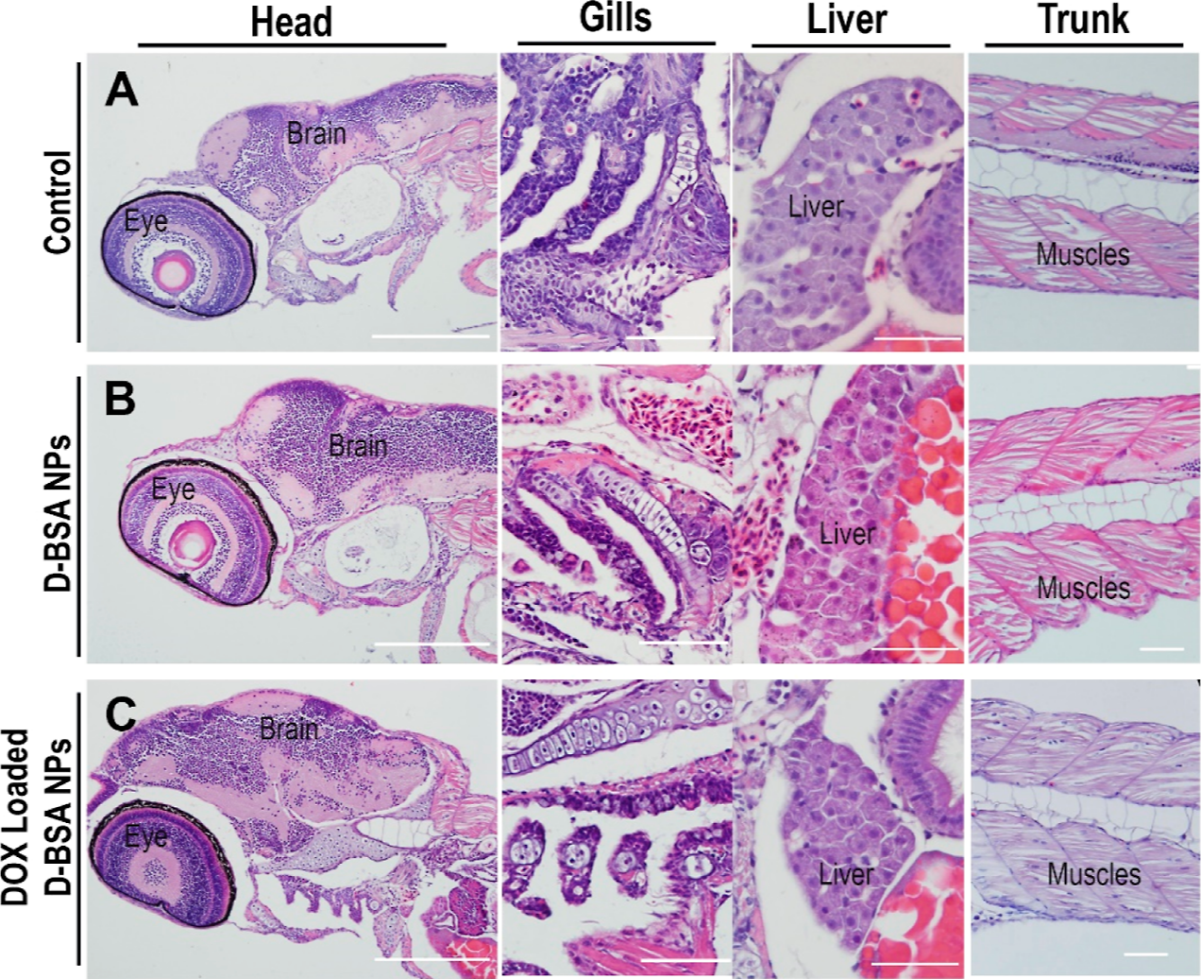
H&E-stained paraffin section images of xenograft larvae at 1 day post-NP injection, from (A) uninjected control, (B) D-BSA NP injected, and (C) DOX-loaded D-BSA NPs injected. Head sections (first column) show the normal brain and eye structures, section from the gill area (second column) shows undamaged cellular structures, liver sections (third column) show healthy hepatocytes, and hepatic tubules and undisturbed tissue morphology, trunk sections (last column) show normal muscle structures. Scale bars: 100 μm (first column) an 50 μm (second-fourth columns).

On the other hand, the treated larvae did not display any signs of toxicity in the normal morphology. In order to confirm a lack of toxicity to the host tissues, histopathological analysis was conducted. Larvae that were xenografted were injected with D-BSA NPs, DOX-loaded D-BSA NPs at 1 dpi and paraffin sections of xenografted larvae were prepared 1 day after NP injection. Tissues were examined after H&E staining, and both NP-injected larvae displayed normal histology when compared to the control larvae that were not injected with NPs (Figure 12). Tissues of uninjected, D-BSA NP- and DOX-loaded D-BSA NP-injected xenografts are shown in four different larval parts. Figure 12 shows that head structures, including the brain and eye, gills, liver and trunk muscles, are intact and normal. The health of the liver is particularly important as it is the major drug detoxifying organ. This finding shows that the synthesized NPs can deliver DOX to a small number of cancer cells present in the zebrafish vasculature, while the healthy tissues remain unaffected in the zebrafish model.

## 4 Conclusions

pH-responsive serum albumin NPs can be prepared with the help of catechol-V(III) complex formation. An average of 15 dopamine (D) molecules containing catechol end groups were conjugated to a BSA protein. Modified D-BSA proteins were desolvated with acetone, and then aggregated proteins were cross-linked using V(III) ions. Tris-catechol-V(III) complex formation was achieved in the pH between 7.4 and 8.0 to prepare D-BSA NPs. The advantage of using V(III) ions over Fe(III) ions, which is commonly used in cross-linking mechanisms, is that tris-catechol-V(III) complex formation can be predominated at relatively lower pH values compared to the pH value of tris-catechol-Fe(III) complex formation (pH 9.0). Therefore, working with V(III) at lower pH values prevents oxidation of catechol groups and leads to the formation of uniformly sized NPs with an average of 253 nm.

pH-sensitive catechol-V(III) bonds allow NP formation at pH between 7.4 and 8.0 but degrade them at acidic pH values: 5.5 and 4.2. Tris-catechol-V(III) coordination provides compact D-BSA NPs in the PBS buffer at pH 7.4. However, the maximum intensity of the particle size distribution obtained from DLS increased from 210 to 342 and 530 nm if the pH values of PBS buffer medium changed from 7.4 to 5.5 and 4.2, respectively, because of opening structures. Moreover, decreasing the pH value increased the PDI values of the NPs from 0.28 to 0.41 and 0.59 at pH 7.4, 5.5, and 4.2, respectively. The drug release was also studied to show the pH-responsive property of the obtained NPs. DOX-loaded D-BSA NPs exhibited different DOX release patterns depending on the pH of the medium. In the first 8 h, the drug release rate was slow and limited to 31% at pH 7.4, while it reached to 42 and 75% at pH 5.5 and 4.2, respectively. Also, total DOX releases reached to 51, 76, and 95% at pH values 7.4, 5.5, and 4.2, respectively, at the end of 80 h.

To sum up, obtaining smaller NPs with a lower PDI value, and having slow and limited drug release could be explained by the existence of tris-catechol-V(III) complexes at pH 7.4. On the other hand, decreasing pH to 5.5 and 4.2, the size of NPs and the PDI value of measurement increased, and the drug release became faster and higher due to the conversion of tris-complexes to bis- and mono-complexes, respectively.

Cell entry of NPs was found to increase in a time-dependent manner. Interestingly, entry of NPs into cancer cells were found to be much more efficient than that of non-cancerous HEK293T cells under in vitro conditions. Another intriguing observation was the better uptake of DOX-loaded NPs into cells when compared to unloaded D-BSA NPs, which may be due to the difference in surface charge and/or chemistry. Cell viability test showed that synthesized D-BSA NPs are not toxic to cancerous or healthy cells, an observation that was confirmed by the apoptosis assay. While the tested cells were sensitive to free DOX, the cell death was increased when DOX-loaded D-BSA NPs were used. This indicated that the easy cell uptake of D-BSA NPs facilitated DOX delivery into cells and improved effectiveness. Finally, the efficiency of D-BSA NPs for drug delivery in vivo was shown with a zebrafish larval xenograft model. Here, the NPs quickly targeted the CTCs in zebrafish vasculature and caused a 30% decrease in the MCF-7 cell number within a day of treatment. The NPs did not cause toxicity to the zebrafish organs as assessed by histopathological analysis of whole larval body.

Being the most abundant plasma protein serum albumin-derived NPs are expected to be fully biocompatible. Biodegradability by the trypsin enzyme over 30 h and lack of hemolytic activity on murine RBCs support the biocompatibility of D-BSA NPs and support the findings in the zebrafish model. Overall, these findings indicate a high performance of the synthesized D-BSA NPs with low toxicity and high efficiency in drug delivery. The use of human serum albumin (HSA) would be required to produce an end product that can be used in clinic. To this end, future studies should be conducted to produce HSA NPs, confirm efficiency, cancer selectivity, and safety in higher preclinical models.

## Supporting Information Available

Zeta potentials and DLS results of the dopamine, BSA, D-BSA, and D-BSA NPs, EPR study of TEMPOL-BSA binding, XRD result of NPs, pictures of obtained materials and hemolysis assay, and flow cytometry results (PDF)
