# Supplementary material for: Dopamine-Conjugated Bovine Serum Albumin Nanoparticles Containing pH-Responsive Catechol-V(III) Coordination for In Vitro and In Vivo Drug Delivery
Source: Biomacromolecules. 2023 Jul 14;24(8):3603–18. doi: 10.1021/acs.biomac.3c00363 (PMC10428161; doi:10.1021/acs.biomac.3c00363)
Supplement: Supplementary file 1 — bm3c00363_si_001.pdf [file bm3c00363_si_001.pdf]

Supporting Information

Dopamine conjugated bovine serum albumin nanoparticles containing pH responsive catechol-V(III) coordination for in vitro and in vivo drug delivery

*Eda Argitekin‡, Esra Ersoz-Gulseven‡, Gulcin Cakan-Akdogan, Yasar Akdogan**

Materials Science and Engineering Department, Izmir Institute of Technology, Turkey

Izmir Biomedicine and Genome Center, Izmir, Turkey

Department of Medical Biology, Faculty of Medicine, Dokuz Eylul University, Izmir, Turkey

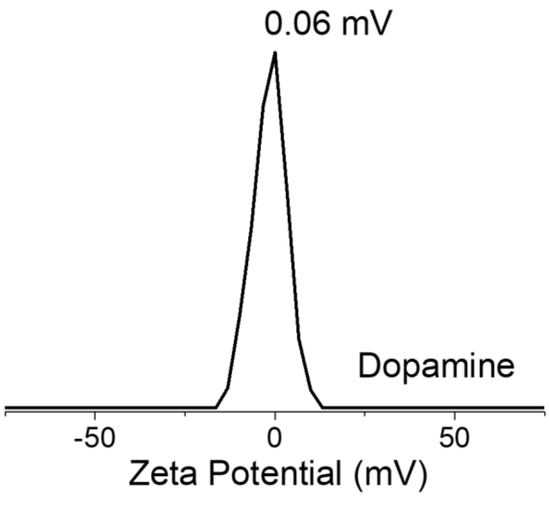

Figure S1. Zeta potential of 1.7 mg/mL of dopamine in water.

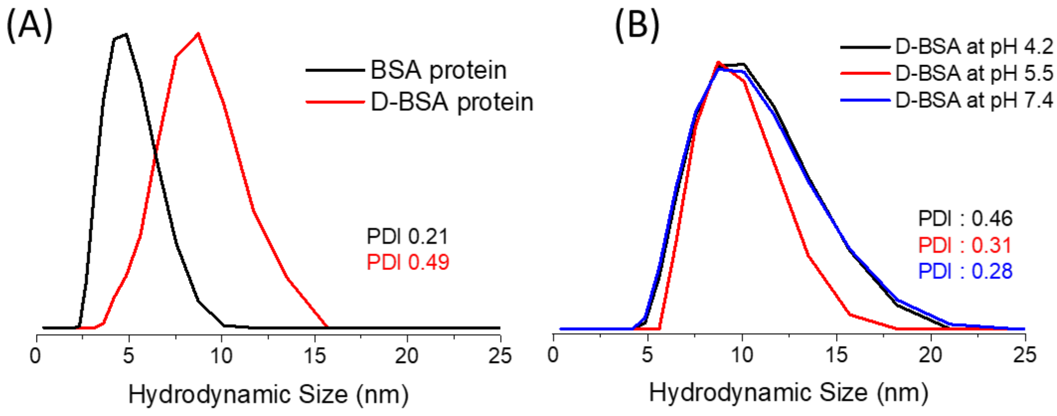

Figure S2. (A) DLS results of hydrodynamic size distributions of BSA (black) and D-BSA (ref)proteins. (B) DLS results of hydrodynamic size distributions of D-BSA proteins at different pH values.

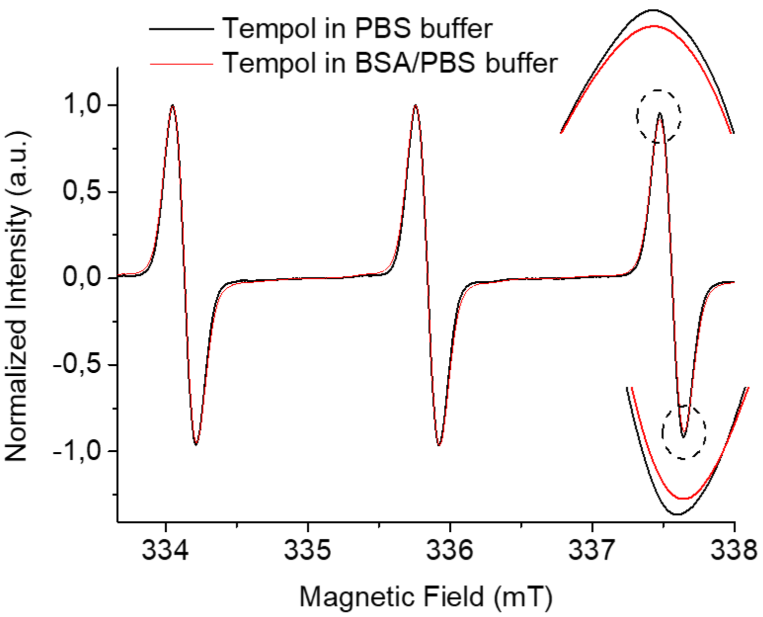

Figure S3. EPR spectra of TEMPOL in 0.05 M PBS buffer solution (black) and in 0.5 mM BSA / PBS buffer (0.05 M) solution (red) at pH 7.4.

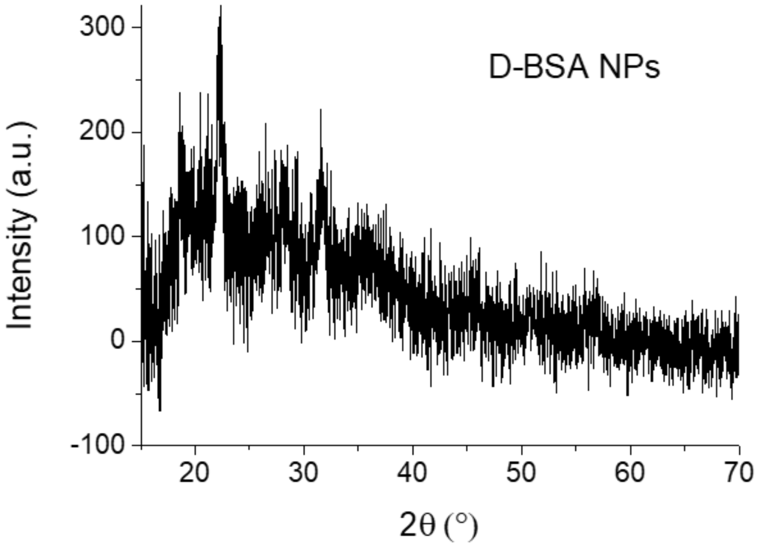

Figure S4. The XRD spectrum of D-BSA NPs.

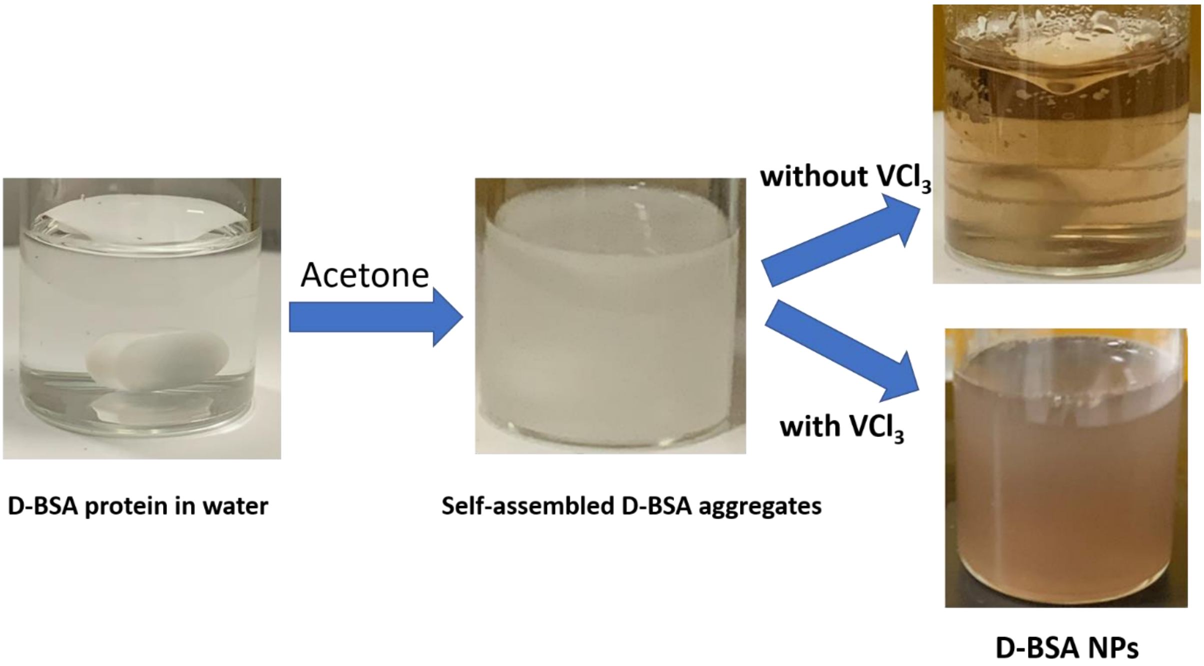

Figure S5. The pictures of D-BSA protein before and after acetone addition in water. Upon addition of VCl_3, D-BSA NPs were formed and thus colloidal dispersion was observed. But, without VCl_3, a transparent solution with precipitation on the vial surface was observed.

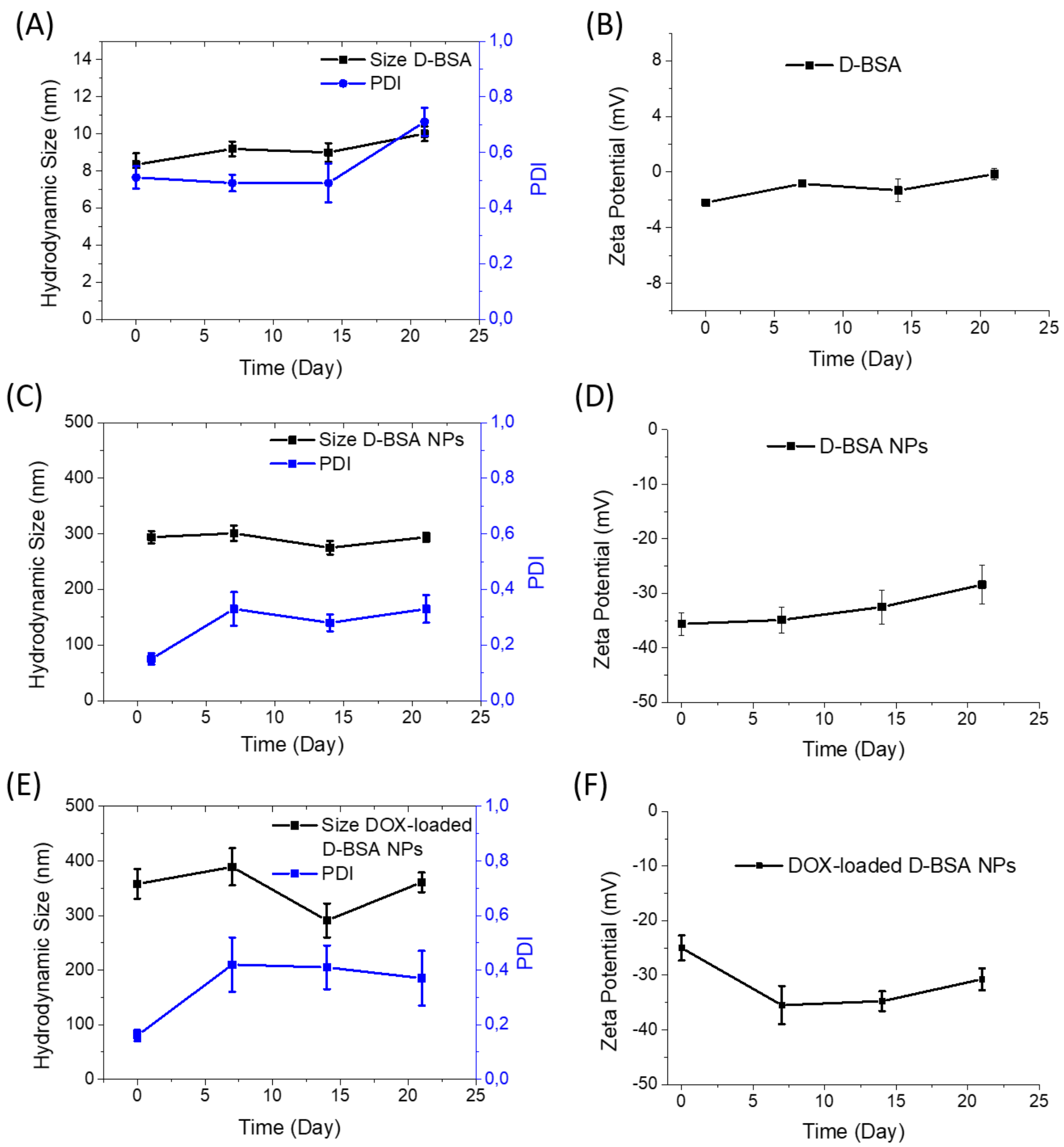

Figure S6. DLS results of hydrodynamic size distributions and zeta potentials of D-BSA protein (A, B), D-BSA NPs (C, D) and DOX-loaded D-BSA NPs (E, F) in water within 21 days.

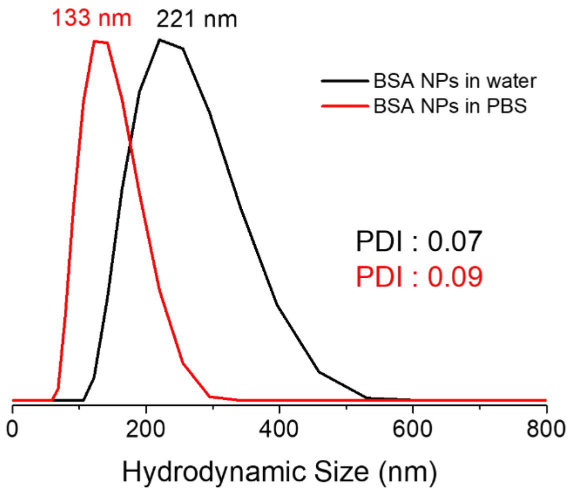

Figure S7. (A) DLS results of hydrodynamic size distributions of BSA NPs in water (black) and in PBS (red). The protocol for BSA NPs preparation by Sozer et al. was followed to prepare BSA NPs using glutaraldehyde as crosslinker after desolvation with ethanol.

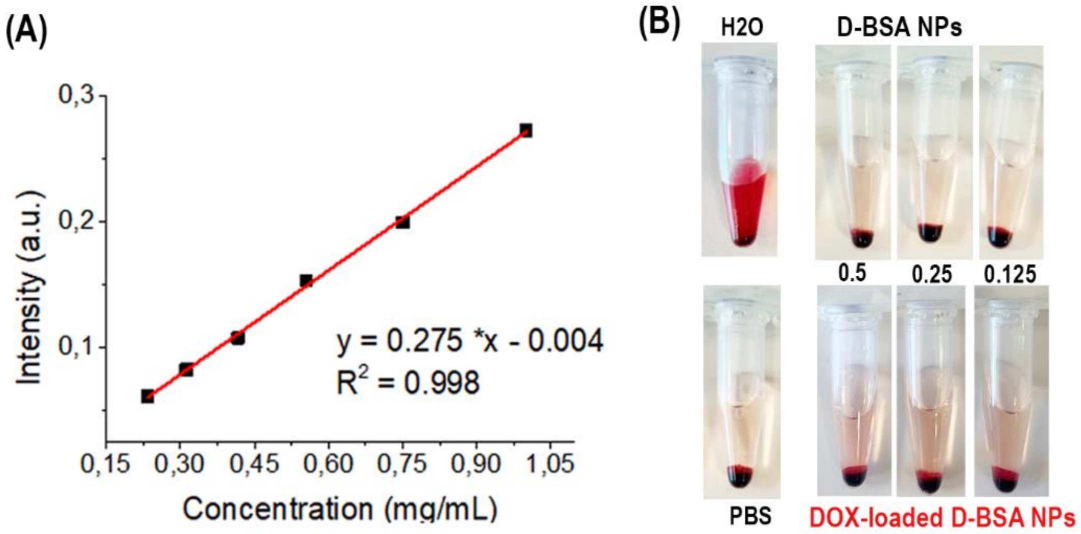

Figure S8. (A) Calibration curve obtained from the turbidity results of D-BSA proteins at different concentrations at 565 nm using UV-Vis absorption spectroscopy. (B) Images of RBC supernatants after hemolytic test. Water used as positive control resulted lysis of RBCs and the supernatant is red which can be detected at 540 nm absorbance. Supernatants after D-BSA NPs and DOX-loaded D-BSA NPs treatment are similar to the supernatant of negative control PBS treated RBCs.

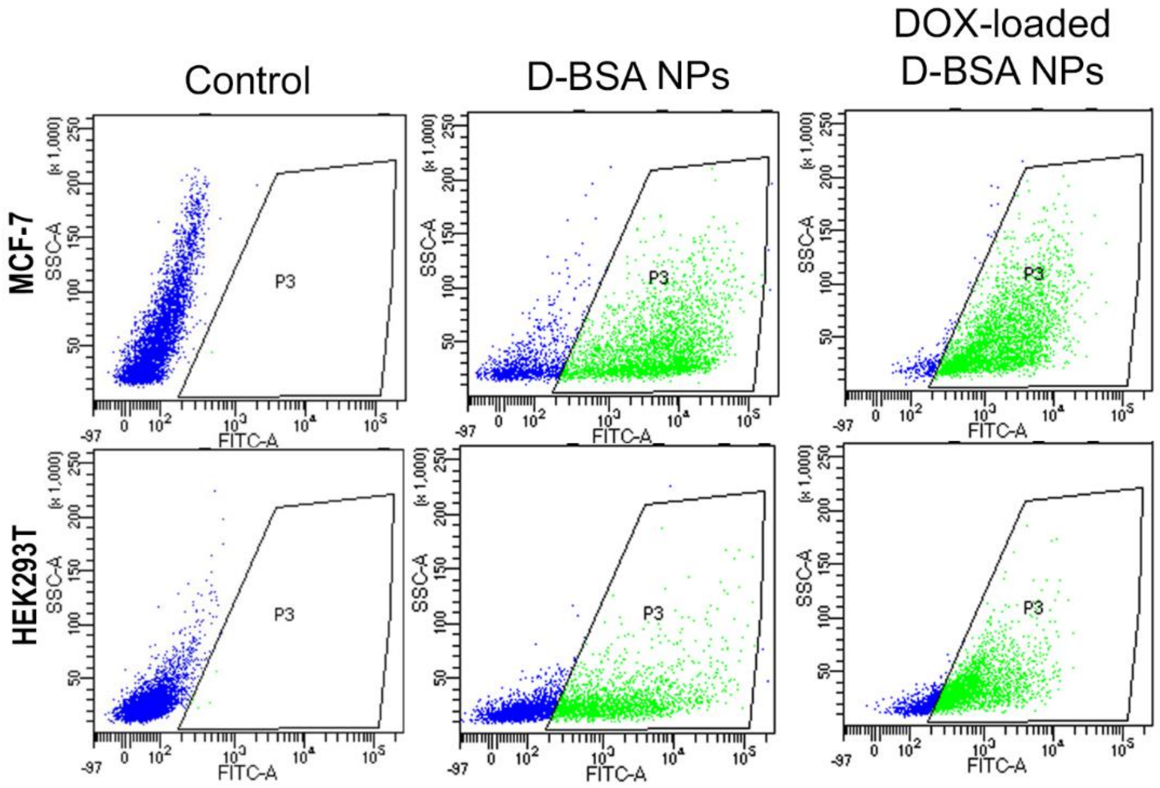

Figure S9. Flow cytometry result of cellular uptake after 24 hours incubation with FITC labeled unloaded and DOX-loaded NPs. FITC positive cells (green) are detected in section P3 of the plot, and FITC negative cells are displayed in blue. Better uptake by MCF-7 cells is observed. Better uptake of DOX-loaded NPs is observed in both cell types.

Reference

1. Sozer, S. C.; Egesoy, T. O.; Basol, M.; Cakan-Akdogan, G.; Akdogan, Y. A simple desolvation method for production of cationic albumin nanoparticles with improved drug loading and cell uptake. *J. Drug Deliv. Sci. Technol.* **2020**, 60, 101931.
